# DNA methylation, transcriptome and genetic copy number signatures of diffuse cerebral WHO grade II/III gliomas resolve cancer heterogeneity and development

**DOI:** 10.1186/s40478-019-0704-8

**Published:** 2019-04-25

**Authors:** H. Binder, E. Willscher, H. Loeffler-Wirth, L. Hopp, D. T. W. Jones, S. M. Pfister, M. Kreuz, D. Gramatzki, E. Fortenbacher, B. Hentschel, M. Tatagiba, U. Herrlinger, H. Vatter, J. Matschke, M. Westphal, D. Krex, G. Schackert, J. C. Tonn, U. Schlegel, H.-J. Steiger, W. Wick, R. G. Weber, M. Weller, M. Loeffler

**Affiliations:** 1 Interdisciplinary Centre for Bioinformatics, Universität Leipzig, Härtelstr. 16–18, 04107 Leipzig, Germany; 2 Hopp Children’s Cancer Center Heidelberg (KiTZ), Im Neuenheimer Feld 430, 69120 Heidelberg, Germany; 3 Pediatric Glioma Research Group, German Cancer Research Center (DKFZ), Im Neuenheimer Feld 280, 69120 Heidelberg, Germany; 4 Division of Pediatric Neurooncology, German Cancer Consortium (DKTK), German Cancer Research Center (DKFZ), Im Neuenheimer Feld 280, 69120 Heidelberg, Germany; 5 Department of Pediatric Oncology, Hematology and Immunology, Heidelberg University Hospital, Im Neuenheimer Feld 430, 69120 Heidelberg, Germany; 6 Institute for Medical Informatics, Statistics and Epidemiology, University of Leipzig, Härtelstraße 16–18, 04107 Leipzig, Germany; 7 Department of Neurology, University Hospital and University Zurich, Frauenklinikstrasse 26, 8091 Zurich, Switzerland; 8 Clinic for Neurosurgery, Tübingen University Hospital, Hoppe-Seyler-Straße 3, 72076 Tübingen, Germany; 9 Division of Clinical Neurooncology, Department of Neurology, University Hospital Bonn, Bonn, Germany; 10 Institute of Neuropathologie, University Clinic Hamburg-Eppendorf, Martinistraße 52, 20246 Hamburg, Germany; 11 Department of Neurosurgery, University Clinic Hamburg-Eppendorf, Martinistraße 52, 20246 Hamburg, Germany; 12 Department of Neurosurgery, Technical University Dresden, Fetscherstraße 74, 01307 Dresden, Germany; 13 Department of Neurosurgery, Ludwig Maximilians University Munich and German Cancer Consortium (DKTK), partner site Munich, Marchioninistraße 15, D-81377 Munich, Germany; 14 Department of Neurology, University Hospital Knappschaftskrankenhaus Bochum-Langendreer, In der Schornau 23–25, 44892 Bochum, Germany; 15 Clinic for Neurosurgery, University Düsseldorf, Moorenstr. 5, 40225 Düsseldorf, Germany; 16 Clinical Cooperation Unit Neurooncology, German Cancer Consortium (DKTK), German Cancer Research Center (DKFZ), Im Neuenheimer Feld 280, 69120 Heidelberg, Germany; 17 Neurology Clinic and National Center for Tumor Diseases, University Hospital Heidelberg, Im Neuenheimer Feld 400, 69120 Heidelberg, Germany; 18 Department of Human Genetics, Hannover Medical School, Carl-Neuberg-Str. 1, 30625 Hannover, Germany

**Keywords:** Glioma, Molecular subtypes, DNA methylation, Epigenetics, Astrocytoma, Tumor microenvironment, Cellular composition, Prognosis

## Abstract

**Background:**

Diffuse lower WHO grade II and III gliomas (LGG) are slowly progressing brain tumors, many of which eventually transform into a more aggressive type. LGG is characterized by widespread genetic and transcriptional heterogeneity, yet little is known about the heterogeneity of the DNA methylome, its function in tumor biology, coupling with the transcriptome and tumor microenvironment and its possible impact for tumor development.

**Methods:**

We here present novel DNA methylation data of an LGG-cohort collected in the German Glioma Network containing about 85% *isocitrate dehydrogenase (IDH)* mutated tumors and performed a combined bioinformatics analysis using patient-matched genome and transcriptome data.

**Results:**

Stratification of LGG based on gene expression and DNA-methylation provided four consensus subtypes. We characterized them in terms of genetic alterations, functional context, cellular composition, tumor microenvironment and their possible impact for treatment resistance and prognosis. Glioma with astrocytoma-resembling phenotypes constitute the largest fraction of nearly 60%. They revealed largest diversity and were divided into four expression and three methylation groups which only partly match each other thus reflecting largely decoupled expression and methylation patterns. We identified a novel G-protein coupled receptor and a cancer-related ‘keratinization’ methylation signature in in addition to the glioma-CpG island methylator phenotype (G-CIMP) signature. These different signatures overlap and combine in various ways giving rise to diverse methylation and expression patterns that shape the glioma phenotypes. The decrease of global methylation in astrocytoma-like LGG associates with higher WHO grade, age at diagnosis and inferior prognosis. We found analogies between astrocytoma-like LGG with grade IV *IDH*-wild type tumors regarding possible worsening of treatment resistance along a proneural-to-mesenchymal axis. Using gene signature-based inference we elucidated the impact of cellular composition of the tumors including immune cell bystanders such as macrophages.

**Conclusions:**

Genomic, epigenomic and transcriptomic factors act in concert but partly also in a decoupled fashion what underpins the need for integrative, multidimensional stratification of LGG by combining these data on gene and cellular levels to delineate mechanisms of gene (de-)regulation and to enable better patient stratification and individualization of treatment.

**Electronic supplementary material:**

The online version of this article (10.1186/s40478-019-0704-8) contains supplementary material, which is available to authorized users.

## Introduction

Diffuse WHO grade II and III glioma (in short lower grade glioma, LGG) describe an almost fatal disease of young adults. These tumors share a more indolent course compared with high-grade IV gliomas (glioblastoma, GBM). Recent work has proposed a classification of glioma based mainly on two genetic markers, namely absence or presence of isocitrate dehydrogenase 1 and 2 (*IDH*) mutation and of codeletion of chromosome arms 1p and 19q (codel), overriding histology [14, 37, 45, 56, 66]. *IDH*-mut codel tumors with mostly oligodendroglial histology are associated with the best prognosis; *IDH*-mut non-codel tumors with mostly astrocytic histology are associated with intermediate outcome; and *IDH*-wt, with mostly higher WHO grade (III or IV) tumors are associated with poor prognosis [51, 65]. Besides genetic factors (DNA-)methylation has emerged an important regulator of gene transcription, and its role in tumorigenesis has become a topic of considerable interest [11, 33]. A number of studies have reported alterations of DNA methylation in gliomas [6, 7, 9, 12, 31, 35, 38, 47, 55].

*IDH* mutations occur early in gliomagenesis in the vast majority of WHO grade II and III gliomas. They change the function of the *IDH* enzymes, causing them to produce 2-hydroxyglutarate (2HG), an oncometabolite that represses DNA demethylation [63] and, in consequence, leads to genome wide DNA-hypermethylation subsumed as glioma-CpG island methylator phenotype (G-CIMP) [47]. Whole genome methylation studies have revealed that G-CIMP gliomas split into subgroups differing in the Chr. 1p/19q codeletion status and the total level of methylation [8] where decreased methlylation associates with worse survival and increased risk for recurrence [12] and possibly reflects a global DNA demethylation shift of progressing G-CIMP-tumors. Both, genetic and epigenetic events can drive progression of gliomas leading to nearly identical phylo (epi-)genetic relations [40]. Moreover, recent studies reported continuous phenotypic drifts along a proneuronal-to-mesenchymal axis in *IDH*-wild type GBM associated with increasing therapy resistance that contradict a major role of genetic aberrations as drivers of essential tumor characteristics such as resistance [54] and that are linked to drifts in DNA methylation [32] and the cellular composition of the tumor microenvironment [62]. We ask if similar mechanisms can be identified also in *IDH* mutated LGG.

In general, deregulation of cell functions in cancer is encoded in both the genome and epigenome which underscores the importance of epigenetic analyses in parallel to the discovery of transcriptomics and genetics. Current analyses have not yet clarified the relationships between the methylome and transcriptome. In LGGs about 84% of all cases carry *IDH*-mutation with about 35% of them carrying an additional Chr. 1p/19q-codeletion, which enables studying phenotypic variability of the transcriptomes and methylomes especially of these genomic strata.

Our previous expression profiling of grade II and III primary glioma from a prospective German Glioma Network (GGN) cohort revealed rich heterogeneity of their transcriptomes which were only partially linked to the genomic features [65]. For this study transcriptomic and genetic data of the 137 lower grade glioma samples from the GGN cohort were complemented by new (DNA-)methylation data of 122 matched tumors of the same cohort which enables a combined analysis aimed to study DNA methylation as a shaping factor of glioma heterogeneity. Here we perform molecular subtyping which has emerged as an important concept to describe glioma heterogeneity and to better understand the biology of this devastating disease. We show that genomic, transcriptomic and methylation data provide partly overlapping but also distinct molecular subgroups, suggesting that different omics-views provide complementary and partly independent information about modes of gene-regulation [26, 57] with potentially different prognostic and therapeutic relevance. We aimed at characterizing the functional context of these different modes with special emphasis on the cellular composition of the tumors and their microenvironment and on possible impact for tumor development from lower grade to higher grade gliomas.

## Materials and methods

### Patients, tumors and data

The GGN study of WHO grade II or III gliomas (LGG) was described previously [65]. For this previous study, we had analyzed tumors of 137 patients by array-CGH, Affymetrix chip-based gene expression and candidate gene analyses (see [65] and Additional file 1: Figure S1). All tumors were subjected to central pathology review and classified according to the 2016 WHO classification of tumors of the central nervous system [37]. For the present study, molecular characterization was supplemented by array-based DNA methylation data (Illumina 450 K arrays) of 122 patient-matched tumors of the GGN cohort (Additional file 2: Table S1).

### Expression, CGH data and DNA methylation analyses

Expression and array CGH data were processed as described in [65]. For genome-wide assessment of DNA methylation glioma samples were arrayed using the Illumina HumanMethylation450 BeadChip according to the manufacturer’s instructions at the DKFZ as described previously [55]. A verification set of WHO grade II and III gliomas was taken from the TCGA repository including gene expression and DNA methylation data (Additional file 3: Table S3). Gene expression data were corrected for background noise, calibrated, quantile-normalized and transformed into log10-scale, as described in [65]. CpG IDs were mapped to the promoter region of each gene ranging from 2 kb upstream to 200 bp downstream of the transcription start site using RefSeq mRNA annotation. DNA methylation beta-values of the respective CpGs were averaged to get one mean methylation beta-value for each gene promoter available. Genes located on Chr. X and Y were excluded from analyses. For an alternative analysis we also integrated CpG methylation over enhancer and gene body regions (see below).

### Bioinformatics analysis

Gene expression and DNA methylation data were centralized and then analyzed after dimension reduction to metagenes using self-organizing map (SOM) machine learning [67]. As a result, each tumor tissue is characterized by the expression/methylation values of 2500/900 metagenes. Downstream analysis tasks including class discovery, visualization and knowledge mining using gene set analysis were performed with the R-package ‘oposSOM’ [36]. Unsupervised class discovery of expression and methylation subgroups was performed independently in metagene space by using maximum spanning graph-partitioning [65] followed by iterative maximization of the sample similarity score until convergence as described before [34]. For gene set profiling we applied the gene set Z-score (GSZ) metrics to estimate the mean differential expression of the set genes in each sample compared with their mean expression levels averaged over all samples in units of the respective standard deviation [58]. We considered gene sets related to biological processes (BP), of the gene ontology (GO) classification, and standard literature sets and literature sets curated by our group [68]. Immune cell composition of the tumor biopsies were estimated from the expression data using the program CIBERSORT [46].

## Results

### WHO grade II/III gliomas split into eight expression and six methylation subtypes

**Fig. 1:**
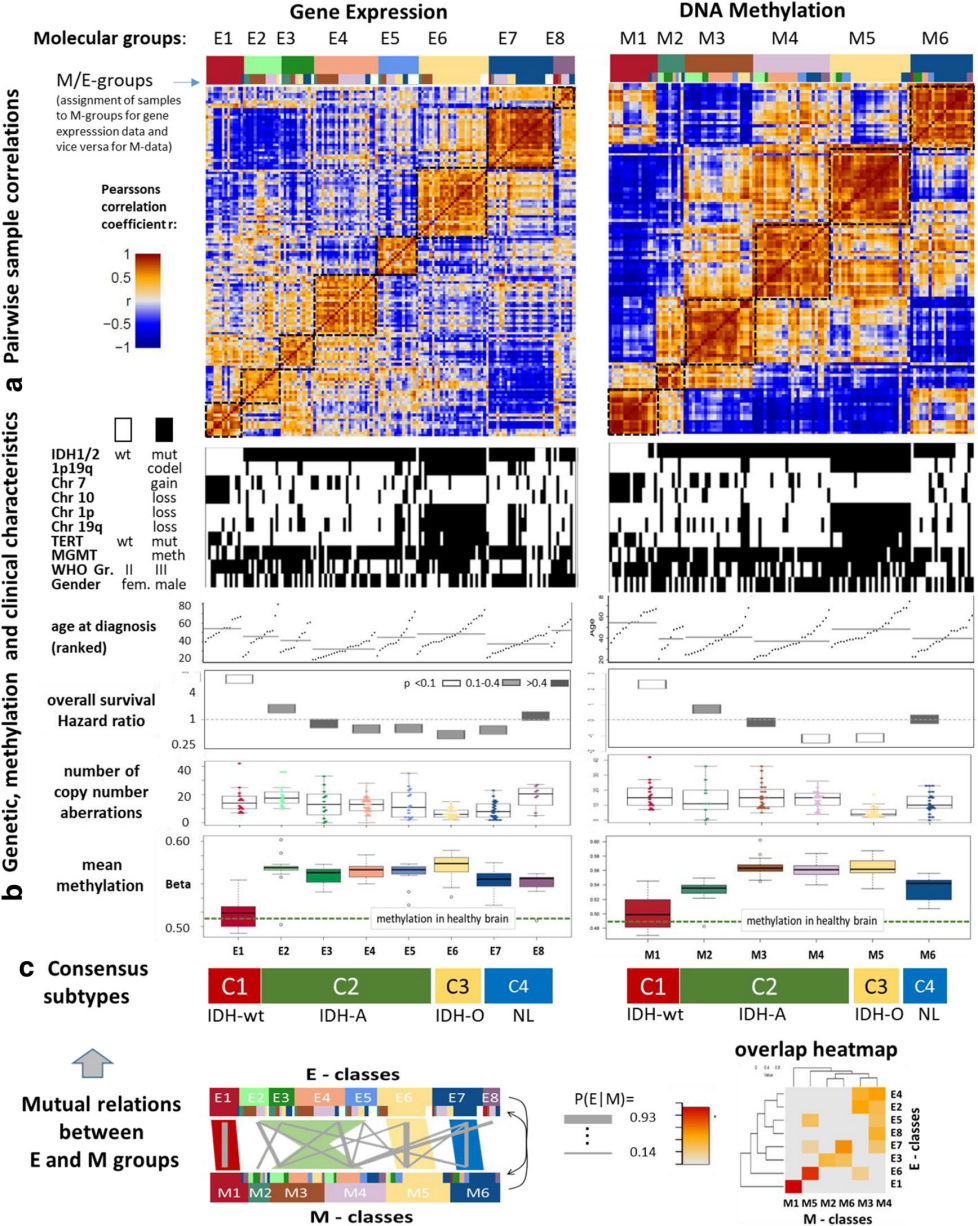
Characteristics of molecular subtypes of glioma. Samples were grouped into gene expression groups E1 – E8 (E-classes) or DNA methylation groups M1 – M6 (M-classes) using the sample expression and methylation data, respectively. A) The pairwise sample correlation heatmaps visualize the correlation coefficient between all pairwise combinations of sample-portraits. Intra-class similarities between samples are evident as brown quadratic areas along the diagonal while inter-class relations are seen either as brown or blue off-diagonal regions for positively and negatively correlated data landscapes, respectively. B) Genetic, methylation and clinical characteristics (see text). C) We sorted samples in each E-group according to their M-group membership and in each M-group according to their E-group membership to better recognize pattern due to methylation and expression effects, respectively (see the two color bars above the heatmap). The color code for molecular groups are used throughout the paper. Mutual relations between the E- and M-groups were estimated based on mutual memberships of the samples giving rise to four consensus subtypes C1- C4 which are characterized by IDH-wild type astrocytoma-like (IDH-wt), IDH-mutated astrocytoma-like (IDH-A) and oligodendroglioma-like (IDH-O) and a neuronal-like (NL) phenotypes, respectively

Single data type class discovery of gene expression data of 137 WHO grade II/III gliomas and DNA methylation data of patient-matched samples all collected in the German Glioma Network (GGN) provided eight expression subtypes designated as E1 – E8 (E-groups) and six methylation subtypes (M1 – M6, M-groups, Fig. 1a). The subtypes E1 and M1 nearly completely collect *IDH*-wild type tumors mostly with gains on Chr7 and losses of Chr10 representing genetic hallmarks of glioblastomas [5, 51, 65] (Fig. 1b, Additional file 1: Figure S2 and Additional file 1: Table S4 and Additional file 1: Table S5 for sample counts and enrichment analysis). The subtypes E2 – E6 and M2 – M5 nearly exclusively contain *IDH* mutated tumors predominantly without codeletions on Chr1p and Chr19q as genetic hallmarks of astrocytomas while the subtypes E6 and M5 strongly enrich samples with a codeletion on Chr1p and Chr19q as a genetic hallmark of oligodendrogliomas [37]. Gains on Chr7 that are not paralleled by losses on Chr10 are frequently found in E4 and M3 (Additional file 1: Figure S2). A chromosome map of gene expression reveals dose-response effects of all these chromosomal defects (Additional file 1: Figure S3A). We find a relatively high number of aberrations in E2 and a relatively small one in E7/M6 and E5/M4 (Fig. 1b and Additional file 1: Figure S2). Interestingly, a bimodal differential methylation pattern between M1 – M3 (reduced methylation) and M4 – M6 (increased methylation) is detected for the olfactory subgenome [19] which collects genes encoding G-protein coupled receptors (*GPCR*’s) especially on Chr11 (Additional file 1: Figure S3B). The E-groups do not show this clear separation into two entities although the amount of hypomethylated *GPCR*’s increases progressively from E7 to E1 (Additional file 1: Figure S3B). A similar bimodal methylation patterns is found for gene clusters encoding keratin intermediate filament proteins on Chr12 and Chr17 in the M-groups (Additional file 1: Figure S3B). We compared the mean methylation levels of the promoter regions as used throughout this work with those of enhancer and gene body regions and found similar methylation patterns on average (Additional file 1: Figure S4), which suggests that aberrant methylation affects widespread genomic regions.

### Consensus subtypes assign to astrocytoma-like, oligodendroglioma-like and neural phenotypes

Detailed analysis of the distribution of samples among the E- and M-subtypes reveals large overlap of tumors and thus correspondence between E1 and M1, E6 and M5 and also between E7 and M6 (Fig. 1c) while E2 – E5 intermix with M2 – M4 with partial correspondence between E3 and M2. Based on these results we define the consensus classes C1 – C4 where C1, C3 and C4 represent classes with almost one-to-one mutual correspondence between the expression and methylation subtypes. With a nearly exclusive content of *IDH*-wild type tumors in C1 (100% in E1 and 87.5% in M1) and of *IDH*-mutated *and* Chr1p/19q codeleted tumors in C3 (92% in E6 and 100% in M5) these subtypes show clear genetic characteristics that assign them to expression and methylation phenotypes of *IDH*-wild type astrocytoma-like (IDH-wt) and to oligodendrogioma-like (IDH-O) resemblance, respectively [37]. In contrast, C2 is a more heterogeneous group regarding the correspondence between the E- and M-classes. It collects predominantly *IDH* mutated tumors (more than 97% in C2) almost always without Chr1p/19q codeletions (85% for E-groups and 90% for M-groups) and without alterations on Chr7 and Chr10 (95%) (see also Additional file 1: Table S4) which assigns C2 to gliomas of *IDH*-mutant astrocytoma-like resemblance (IDH-A) [37]. Nevertheless, a minority of about 15% of all *IDH*-mutant and Chr.1p/19q codeleted oligodendrogliomas are in C2 (12.5% in the E-groups and 17% in the M-groups) mostly because of a decreased methylation level of the *GPCR* subsumed in the olfactory subgenome that contrasts them compared with the majority of 67% oligodendrogliomas in C3/IDH-O (60% in E6 and 75% in M5) and also in C4 (15% / 8%, Additional file 1: Figure S3B). The consensus subtype C4 collecting E7, E8 and M6 constitutes mixtures of tumors with genetic characteristics present in all remaining subtypes. We assign specimens with reduced tumor cell content to C4 based on the observations that the mean number of copy number aberrations on Chr7 and Chr10 in E1 and on Chr1p and Chr19q in E6 is reduced for samples in E7, respectively (Additional file 1: Figure S5). Additionally, C4 shows a healthy brain functional context, e.g. related to synaptic transmission (see below). Hence, the expression and methylation landscapes of the glioma subtypes are shaped in first instance by the underlying key genetic defects in agreement with a recent classification of LGGs [56]. However, we also found a large degree of inter-tumoral heterogeneity of expression and methylation phenotypes that considerably modulates this genetic picture as illustrated by means of sample-similarity nets based either on the gene expression or on the methylation data (Additional file 1: Figure S7). This uncertainty obviously results, among other factors (such as tumor purity and composition), from the multidimensional nature of the transcriptomes and methylomes, e.g. from the combination of different G-CIMP- and *GPCR*-methylation patterns, from the lack of a clear-cut one-to-one relation between many of these phenotype-dimensions and the underlying genotypes.

### The subtypes differ in overall promoter methylation, WHO grade and prognosis

Next, we compared the mean absolute promoter methylation level averaged over all genes and samples of each subtype (Fig. 1). It is low in C1 (*IDH*-wt) and high in C3 (IDH-O) and also C2 (IDH-A), as expected, because these predominantly *IDH*-mutated tumors in C2 and C3 form the CpG hypermethylation phenotype (G-CIMP) [47]. The degree of hypermethylation in M2 is reduced compared with the other *IDH* mutated tumors in C2 and C3 while promoter methylation is on intermediate level in C4 collecting a mixture of *IDH*-mutated (64%) and *IDH*-wt (36%) tumors. Interestingly, the mean methylation level of the subtypes inversely relates to their overall survival hazard ratio (Additional file 1: Figure S6). Worst prognosis of *IDH* wild type LGG compared with *IDH* mutated *and* Chr1p/19q codeleted (best prognosis) and non-codeleted ones (intermediate prognosis) was reported previously [65]. Interestingly, we find a similar, however more subtle inverse trend between methylation and HR in the E- and M-groups collected in C2 (IDH-A) that associates with the accumulation of WHO grade II astrocytic tumors in E4 (58%) and E5 (60%, decreased HR and increased methylation, Additional file 1: Table S4) while grade III tumors accumulate in E3 (100%) and E2 (71%, increased HR and decreased methylation). Enrichment of higher tumor grade III is also found in M2 (78%) and M3 (70%). It is associated with worse prognosis and decreased methylation (Additional file 1: Figure S6). Taken together, our data suggest associations between decaying methylation, increasing WHO grade and HR in *IDH* mutated astrocytoma-like tumors (IDH-A).

### Verification using TCGA data and comparison with previous signatures of gliomas

**Fig. 2:**
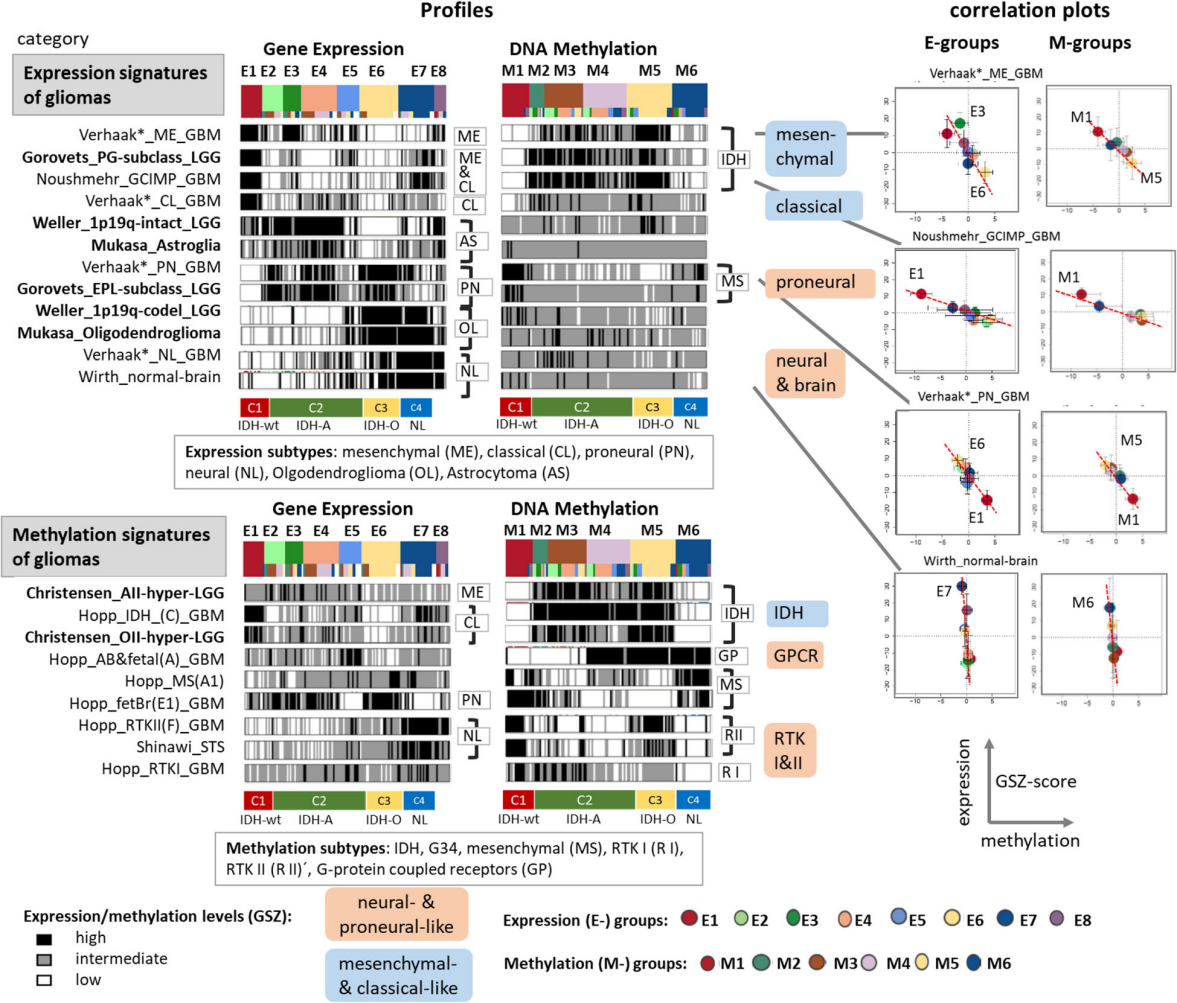
Gene set analysis associates the E- and M-subtypes with previous glioma expression and methylation signatures (see Additional file 1: Table S6 for details). The expression and methylation levels of the signature sets are shown as bar-code profiles where each bar refers to one sample. Correlation plots between expression and methylation levels in GSZ-scale reflect predominantly repressive effects of promoter methylation on the expression of the downstream genes (right part)

The E- and M-subtypes found here were confirmed (except E5) in more than 270 LGGs taken from The Cancer Genome Atlas (TCGA) using a guided SOM-extension method that combines the GGN- and TCGA-data and enables their joint analysis [34] (Additional file 1: Figure S8, Figure S9). Moreover, we selected a series of GBM and lower-grade glioma (LGG) signature gene sets of previous classification schemes and compared them with the subtypes identified herein (Fig. 2 and Additional file 1: Table S6). We found correspondence between our subtypes E1 and partly E3 and signatures of the classical (CL) and mesenchymal (ME) expression subtypes of grade IV gliomas [60], of the pre-glioblastoma (PG) subtype of LGG [20] and of hypermethylated genes of the G-CIMP-phenotype [47]. Signature genes of proneural (PN) GBM [60] and of early-progenitor-like (EPL) LGG [20] show similarities with C2 (IDH-A), with subtle differences between E2, E3 and E4, while neuronal GBM (NL) and healthy brain signatures match to C4 and partly C3 (IDH-O). Interestingly, E3 collects *IDH*-mutated glioma with an inflammatory, mesenchymal-like expression signature. The expression level of most of these signatures sharply change between the E-groups which indicates correspondence between our current classification and those previously described. The analysis of gene sets derived from methylation studies provides analogous results where, e.g., hypermethylation signatures in LGG [9] and the *IDH* subtype of GBM [55] largely agree with the G-CIMP-profile [47] that shows hypermethylation in M2 - M5 (Fig. 2). Oligodendroglial glioma reveal a modified G-CIMP-profile (G-CIMP-O) with enhanced methylation in M5 that closely resembles the RTKII profile [55]. CpG-level marker sets confirm the G-CIMP and G-CIMP-O profiles [49] (Additional file 1: Figure S10). Interestingly, methylation signatures of fetal and adult healthy brain [27] indicated strong similarity with the *GPCR*-signature meaning that the respective genes markedly lose methylation in gliomas, especially in M1 – M3. Overall, methylation signatures from previous studies including those of grade IV GBM [55] indicate similar underlying expression and methylation patterns. Accordingly, the consensus subtype C1 (IDH-wt) possesses pre-GBM (PG) characteristics, C2 (IDH-A) and C3 (IDH-O) are proneural-like (PN) tumors (with E3 showing more mesenchymal-like characteristics) and C4 represents a neural-like (NL) subtype with mixed genetic characteristics of gliomas and expression properties partly resembling those of healthy brain in agreement with [62]. We also compared our subtypes with the epigenetic classes of Ceccarelli et al. [8] making use of CpG-level methylation and epigenetically regulated gene signatures (Additional file 1: Figure S11) and of an extended GGN data set (Additional file 1: Figure S13). Accordingly, M2 tumors reveal resemblance with the GCIMP-low and M3 – M4 tumors with the GCIMP-high classes of Ceccarelli et al. while C1 tumors can be assigned to CL-like (8 cases) and ME-like (6 cases) glioma based on their expression characteristics and to a 40/60% composition of RTKII and MS tumors using their methylation signatures. Most interestingly, the E3 tumors reveal characteristics of pilocytic astrocytomas (PA) which was detected by comparison with the expression patterns of 16 PA samples collected in the GGN. PA-resemblance was established for IDH-wt gliomas by Ceccarelli and colleagues but not for IDH-mut LGG. In summary, almost all E- and M- subtypes could be verified in an independent dataset and by previous glioma signatures where our approach stratifies IDH-mut astrocytomas (C2) in a novel way into three methylation and four expression subtypes which only partly match each to another.

### Functional context and epigenetic signatures

**Fig. 3:**
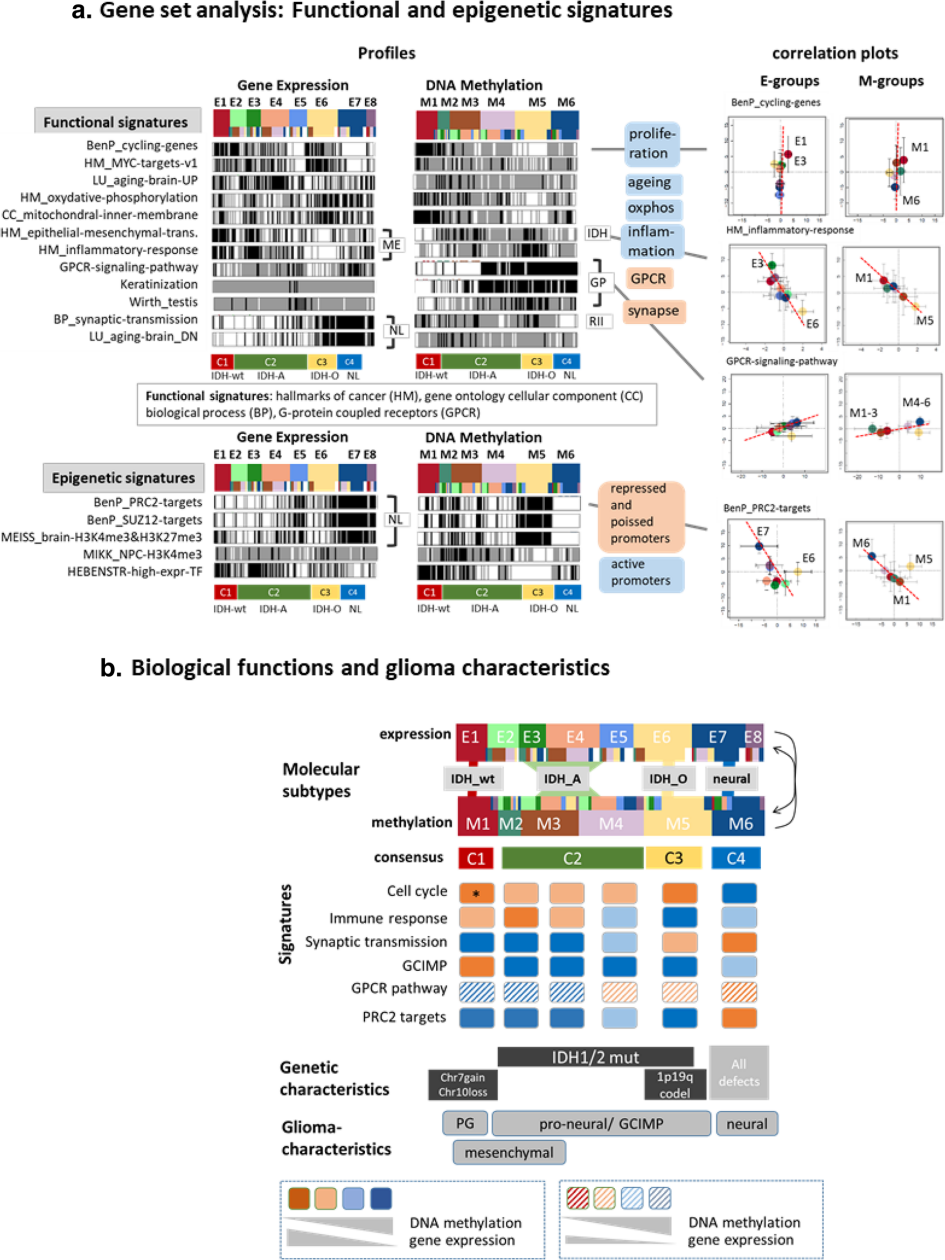
Gene set analysis of functional and epigenetic signatures: **a** Bar-code profiles of expression and methylation levels of functional and epigenetic signatures and the correlation plots of subtype averaged values (see legend of Fig. 2). **b** Schematic overview about the basic functional, genetic and glioma characteristics extracted from the gene-signature analysis

Next we analyzed the functional context of the E- and M-subtypes of our data set (Fig. 3 and Additional file 1: Figures S14- S16). Gene signatures reflecting highly proliferating cells and high levels of oxphos metabolism are strongly expressed in E1 and E6 but weakly expressed in E7 which instead shows activated cell functions of healthy brain such as synaptic transmission. Inflammatory responses and a signature of epithelial-mesenchymal transition (EMT) were high in E3 and to a lesser degree observed in E1 but almost deactivated in C3 (IDH-O). Profiling of a series of metabolic gene sets confirms high oxphos and mitochondrial transcriptional activity in C3 paralleled by decreased glycolysis (Additional file 1: Figure S15) while C2 (IDH-A) is characterized by gained methylation and decreased expression of genes related to fatty acid metabolism, oxphos and mitochondrial functions. Interestingly, E2 seems metabolically deactivated throughout all processes considered while C1 (IDH-wt) shows the opposite trend.

Gene signatures of the ageing brain suggest parallels with inflammatory signatures upregulated in E3/E4. The methylation profiles of all these signatures show mostly anti-correlated patterns compared with the expression profiles (see the right ‘methylation’ part of Fig. 3a and Additional file 1: Figure S14). It indicates that promoter methylation predominantly represses transcription of the respective downstream gene. Gene sets estimating the activity of G-protein coupled receptors (*GPCR*), and of keratinization both show binary methylation patterns with low levels in M1 – M3 and higher levels in M4 – M6 and correlated expression with the inflammatory signature and anti-correlated expression with the signature of synaptic transmission. Signatures related to epidermal cell differentiation and keratinization are prone to hypo-methylation also in other cancers [71]. They are found to tune the balance between stemness and somatic functions [28], to promote EMT-like processes [50] and also can serve as prognostic markers in epithelial cancers [30]. Testis-specific genes are overexpressed in E5. This phenomenon is observed also in other cancers [25] where so-called cancer testis (CT) genes often encode antigens that are thought to be immunogenic in gliomas and particularly in cancer stem cells [16, 18, 73].

Interestingly, also signatures with impact for epigenetic mechanisms of gene regulation reflect pronounced subtype-specific differences (Fig. 3a, part below). Particularly, H3K4me3 marked genes in active promoters of neural progenitor cells (NPC) [42] and transcription factors (TF) associated with high expression levels in a wide collection of cells [24] show low methylation in M5 contrasted by high methylation in M1 and partly in M2 and M3 and thus similar trends as observed for the signatures related to highly proliferating cells and *MYC* targets discussed above. This seems plausible for M5 because highly proliferative cells require promoters activated by demethylation while activation of proliferation genes in M1 requires another mechanism. In contrast, hypermethylation in C3 (IDH-O), and to a less degree in C1 (IDH-wt) and C2 (IDH-A), is observed for targets of the polycomb repressive complex 2 (*PRC2*) in de-differentiated tumor cells [3], for related compounds such as *SUZ12* and *EED* targets and for bivalently H3K4me3 and H3K27me3 marked genes in poised promoter states that are enriched in tumor suppressors [41]. Their suppression via hypermethylation promotes cancer development in gliomas and in other cancer entities [27]. The respective expression and methylation profiles closely resemble those of healthy brain and synaptic transmission thus suggesting their suppression by epigenetics in gliomas. It is known that *PRC2* is required for neuron specification during differentiation and for suppression of a transcriptional program that is detrimental to adult neuron function and which in case of *PRC2* deficiency leads to neurodegeneration via de-repression of bivalent *PRC2* target genes [61, 70]. An analysis of genes in a set of defined chromatin states [15] determined in healthy fetal and adult brain tissues representing different states of brain development [52] further supports the view that suppressor-mechanisms in cellular programs are related to brain development and that genes in repressed states with impact for brain differentiation become hypermethylated in G-CIMP-subtypes and especially in C3 (Additional file 1: Figure S17).

Detailed functional analysis reveals anti-concerted alterations of expression and methylation, which associate with transcriptional activation of cell cycle related biological processes and the decay of neuronal processes such as synaptic transmission especially in C1 and C3 and also with changes of inflammatory characteristics in C2 (Additional file 1: Figure S18). Overall we identified three combined expression-methylation patterns (Additional file 1: Figure S19), namely (i) activating modes were related to proliferation and show increased expression which however associates either with increased (C1/IDH-wt) or decreased (C3/IDH-A) methylation reflecting different driving mechanisms; (ii) deactivating modes which combine decreased expression and increased methylation in all subtypes associated with functions such as synaptic transmission; and (iii) functions related to immune response also showing anti-correlated changes between expression and methylation but an activating effect in C1 and especially E3 and deactivating effect in C3. Hence, degeneration of healthy brain functions in all subtypes, activated proliferation in C3 (IDH-O) and partly inflammation in E3 seem to be affected by anti-correlated DNA-promoter-methylation changes. In summary, the subtypes were characterized by combined alterations of the methylation and expression levels of genes from cellular programs such as proliferation, energy metabolism, immune response and synaptic transmission which associate with repressed and poised chromatin states in healthy brain and their subtype-specific remodeling in gliomas (Fig. 3b).

### Reference to single cell signatures disentangles glioma cell types

**Fig. 4:**
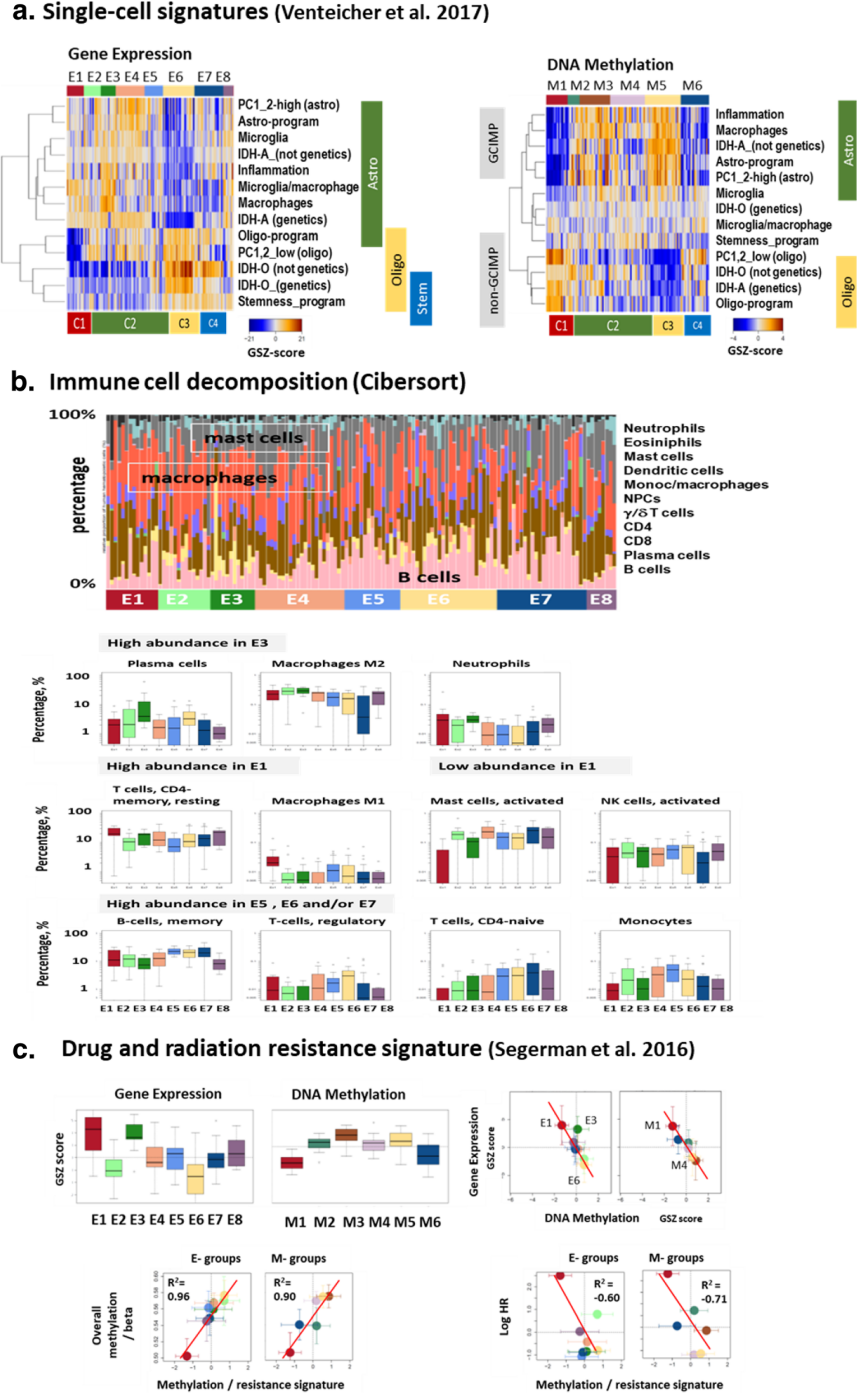
Cell type, micro-environmental immune cell and treatment-resistance characteristics. **a** Heatmaps of expression and methylation levels of single-cell signatures taken from [59] reveal subtype-specific activation of astrocyte-, oligodendrocyte- and stem cell-like characteristics. **b** Digital immune cell-type decomposition of glioma transcriptomes using CIBERSORT [46] (see Fig. S21 for the full set of cells considered) on sample (above) and mean subtype levels for selected leukocyte cells across the expression subtypes. **c** The boxplots of expression and methylation levels of a transcriptomic drug and radiation resistance signature containing 50 genes [54] suggest largest resistance effects in E3 and E1. Expression and methylation levels of the subgroups anti-correlate (right part)

Gliomas are composed of neoplastic and non-neoplastic cells, each of which potentially contribute to cancer formation, progression and response to treatment [22, 62]. Bulk expression and methylation profiles as analyzed in this work average the diverse cell signatures within each tumor, thereby potentially masking critical differences and providing limited insight into cancer cell programs and the effect of the tumor microenvironment (TME). To disentangle this heterogeneity on cellular and TME-levels, we evaluated the expression and methylation degree of a collection of gene signatures taken from recent single cell RNAseq experiments on gliomas [59] in our data (Fig. 4a). We find that C2 gliomas were characterized by relatively high expression levels of benign astrocytes (astro-program, especially in E4), malignant astrocyte-like cells (*IDH*-A signature) and of microglia/macrophages (especially in E3) which all confirm the astrocyte-like phenotype of C2. On the other hand, these signatures are all low in C3 tumors which instead show activated expression of oligodendrocyte-like cells (oligo-program and *IDH*-O signatures), of stemness and of neuronal signature genes where the latter ones are also high in C4 (neural subtype). The expression characteristics associate with almost mirror symmetrical methylation profiles showing either G-CIMP- or anti-G-CIMP characteristics, thus again suggesting regulatory effects of gene promoter methylation on downstream gene expression. A more detailed analysis indicates anti-correlated expression and methylation patterns of the malignant *IDH*-A and *IDH*-O dimensions suggesting that neoplastic transformations in *IDH*-O and *IDH*-A cells are driven by de-methylation of the respective signature genes while cell cycle and microglia/macrophage signatures increase and neuronal, healthy astro- and oligo-program signatures decline with increasing grade (Additional file 1: Figure S20). Overall, C3 tumors share closer similarities with healthy brain functions than C2-gliomas. C2-tumors instead show enhanced expression of macrophages/microglia signatures where microglia are crucial immune cells of the central nervous system and serve as tissue-resident macrophages of the brain [53]. On the other hand, both, C2 (IDH-A) and C3 (IDH-O) tumors are more proliferative compared with neuronal ones (C4). A higher amount of microglia/macrophage cells in astrocytoma and an increasing amount of proliferating cells is known to be a hallmark of higher grade gliomas [22]. In summary, the single cell characteristics reflect the variability of the composition of the tumors regarding healthy and benign astrocyte- and oligodendrocyte-like cells, microglia/macrophage and proliferative stem cell-like constituents in the bulk specimens studied.

### mRNA inferred immune cell components

To further characterize the TME we employed CIBERSORT [46], a computational cell-type deconvolution method that decomposes the glioma-associated stroma into its immunological cellular components using cell-type related expression signatures (Fig. 4b and Additional file 1: Figure S21). We find that M2-macrophages are highly abundant in the astrocytic groups E1 – E4 with highest levels in E3 and E2 opposed by a reduction in monocytes. M2-macrophages play a pro-tumoral and anti-inflammatory role in brain cancer; they promote tissue remodeling and tumor growth [13, 53], particularly in glioma [43], and associate with resistance to radiotherapy in mesenchymal glioblastoma [62]. In contrast, anti-tumoral and pro-inflammatory M1-macrophages are almost absent in all grade LGG subtypes except for the pre-GBM subtype E1. Beyond a dual M1/M2 polarization status, a continuum between M1 and M2 polarization seems to exists in glioma [17] including *IDH*-mut tumors [59] which provides a possible interpretation of the increasing M2 percentage in C2 from E5 to E3. It has been hypothesized that the most aggressive and invasive cells in GBM are neoplastic macrophages arising in fusion hybrids between neoplastic stem cells and macrophages/microglia [29]. The high M2-macrophage abundance in astrocytic gliomas is paralleled by relatively large percentages of neutrophils while increased abundance of M1-macrophages in E1 is accompanied by CD4-resting memory T-cells. The amount of tumor infiltrating CD4+ leukocytes in glioblastoma correlates with tumor progression and presumably relates to tumor angiogenesis [23, 44]. We also found that activated mast cells are relatively abundant in virtually all groups (especially in E4, E5 and E7) except E1. Mast cells were shown to become recruited and ‘educated’ by glioma cells in a glioma grade-dependent manner to reduce stemness, decrease proliferation and migration to induce differentiation of glioma cells [1]. This mechanism seems to apply to early tumor stages of *IDH*-mutated astrocytoma-like gliomas (C2). Interestingly, regulatory T-cells (Tregs) show increased percentage in the oligodendroglioma-like subtype C3, the subtype with lowest immune and inflammatory characteristics, which is in correspondence with the immunosuppressive role of Tregs in glioma [48]. We also make use of immune cell gene signatures taken from [4] to compare their expression and methylation levels (Additional file 1: Figure S21). We find that most of them show high expression especially in E3 and E1 reflecting their accumulation in higher grade astrocytoma. These expression profiles are mostly paralleled by G-CIMP and especially G-CIMP-O methylation profiles which suggest deactivation of immune cell activities in C3 by DNA methylation. Interestingly, the methylation profile of T-cells resembles that of *GPCR*, which suggests a cell specific relation between DNA methylation and gene expression. Hence, the changes of methylation observed originate from both glioma and immune cells, which suggests coupled epigenetic mechanisms during tumor development. Note that DNA methylation in glioma bulk samples was found to be predictive for immune cell infiltration [32]. In summary, digital immune cell deconvolution of the transcriptome reveals that M2-macrophages were enriched in higher grade astrocytomas (E1- E3) while activated mast cells are more abundant in the neuronal subtype (C4), in lower grade astrocytomas (E4- E5) and in oligodendrogliomas (E3) together with immunosuppressive Tregs. Hence, the TME is characterized by marked variations of the immune cell composition that overlays with methylation changes of their genomes which suggests an epigenetically-mediated interplay between development of tumor cells and immune cells in the TME.

### Treatment resistance and senescence signatures associate with methylation

Next, we studied a 50-gene multi-therapy resistance signature, which reflects a continuum of cell phenotypes with increasing resistance against chemo- and radiotherapy paralleled by a proneural-to-mesenchymal shift of their transcriptomes [54]. In our data, we find a profile of this signature showing highest expression in E3 (C2) and C1 and lowest in C3 (Fig. 4c), thus suggesting a gradient of treatment resistance from oligodendroglioma-like to astrocytoma-like tumors with inflammatory characteristics of the TME. The resistance signature resembles the profiles of inflammatory and EMT functional signatures (Fig. 3a) and that of the mesenchymal GBM-subtype (Fig. 2a) in our data. Tumors of the latter type indeed showed enhanced treatment resistance [32, 54, 62]. The methylation profile of the resistance signature reflects G-CIMP characteristics and anti-correlates with the respective expression levels, which suggest a methylation-driven repression mechanism. Interestingly, the methylation profile of the resistance signature strongly correlates with the total methylation level of the tumors (R^2^ > 0.9), which suggests that treatment resistance associates with overall methylation of the tumors. Our data support the view that the methylation profile of the signature anti-correlates with the HR-profile (R^2^ < -0.7, compare also with Fig. 1) showing that worsening of prognosis of astrocytoma-like *IDH*-mutated tumors (C2/IDH-A) associates with de-methylation of the tumors. A GCIMP-low methylation profile, mesenchymal-like expression characteristics and genomic instability was recently found in recurrent gliomas [12] in analogy with the characteristics of M2/E3-tumors reported here (Additional file 1: Figure S2, Figure S12).

A recent model of glioma progression suggests that increased senescence bypass mechanisms proceed in parallel with tumor development and the formation of a pro-inflammatory microenvironment at later phases [2]. We, therefore, studied a signature of genes that contribute to senescence bypass mechanisms by promoter-hypermethylation during aging and tumorigenesis and which associate with cancer risk [69]. These genes become increasingly deactivated in the tumors of the E-groups from E7 to E1, i.e. along the neuronal- proneural to mesenchymal axis (Additional file 1: Figure S23). Their senescence profile resembles those of the PRC2-targets, RTKII-characteristics, ageing and healthy brain signatures, while the methylation profiles of the two latter signatures differ from the former ones regarding methylation of Chr1p/19q-codeleted tumors in C3. Particularly, these tumors show increased methylation of senescence genes accompanied by demethylation and transcriptional upregulation of genes involved in oxphos-metabolism (Fig. 3 and Additional file 1: Figure S15) and/or deactivated inflammatory response. It is assumed that Chr1p/19q-codeleted gliomas (C3) bypass senescence by other mechanisms than Chr1p/19q-non-codeleted tumors [2]. Overall, the LGG-subtypes group along a therapy-resistance signature suggesting that resistance and recurrence are mediated by epigenetics and an inflammatory TME along the proneural mesenchymal-like axis also in LGG accompanied by graded loss of methylation and increased CNV and IDH-wt resemblance. Moreover, astrocytoma-like tumors in C2 seem to develop along this axis as indicated by progressive activation of senescence bypass mechanisms.

## Discussion

### Heterogeneity of WHO grade II and III gliomas

**Fig. 5:**
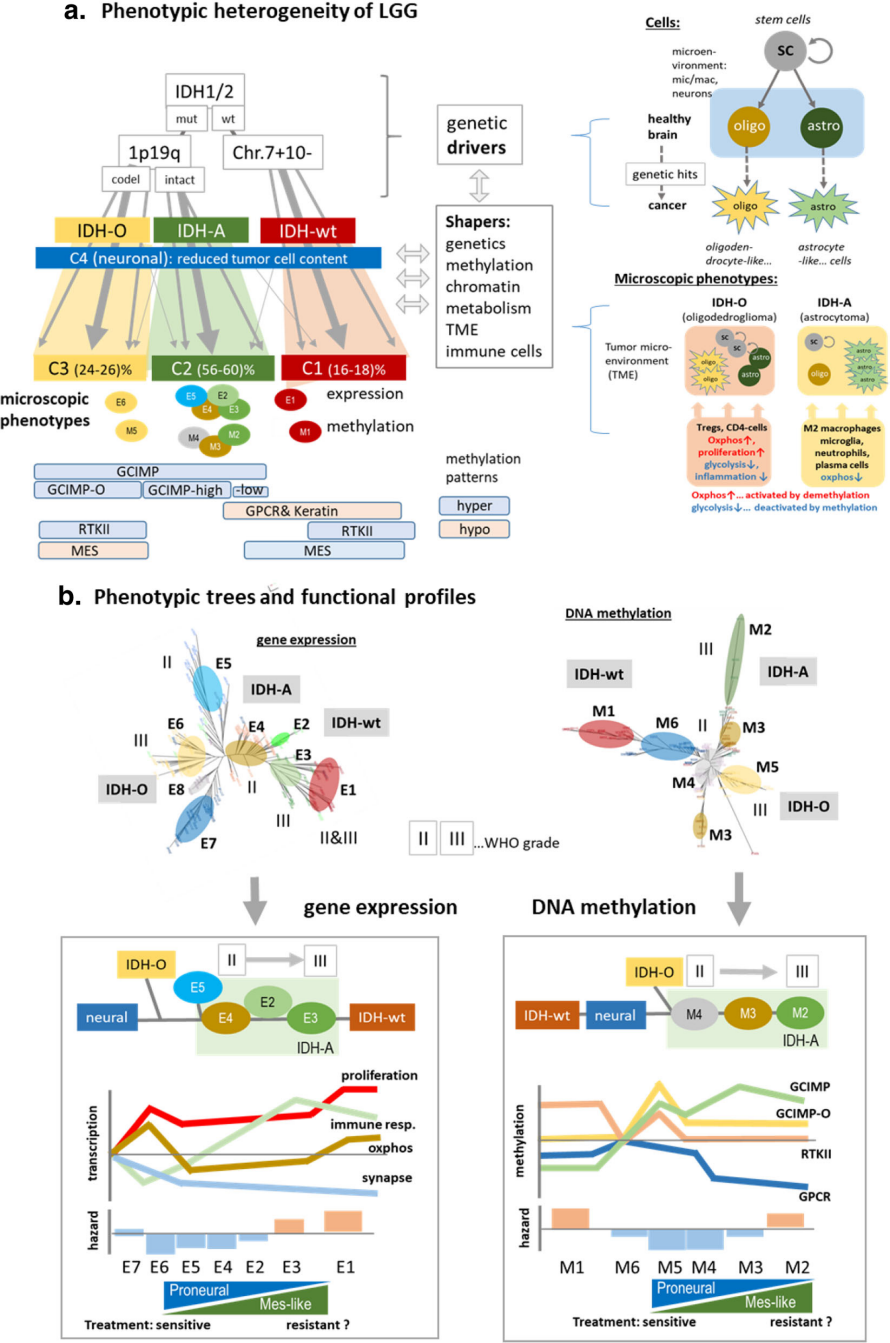
Schematic summary: **a** The major glioma subtypes arise after specific genetic hits. The tumor phenotypes are then shaped by the tumor microenvironment (TME), its cell composition, epigenetics and additional genetic defects. Different methylation patterns develop in a subtype specific fashion upon tumor progression (left part). On a cellular level, astrocyte-like and oligodendrocyte-like gliomas are both primarily composed of proliferating stem cells, oligodendrocytes and astrocytes, however in different amounts, which associates with different immune cell compositions in the TME and metabolic expression signatures, which partly are affected by methylation effects. **b** Phenotypic trees provide similarity relations between the expression and methylation subtypes (top), which were simplified as one-dimensional sequences of subtypes and associated with selected transcriptional programs, methylation patterns and prognosis (bottom)

Our multi-platform transcriptome-methylome-genome study revealed a large molecular heterogeneity of adult diffuse gliomas of WHO grades II and III: we identified eight expression and six methylation subtypes and characterized them in terms of genetic aberrations, functional context, cellular composition, tumor microenvironment and their possible impact on treatment resistance and prognosis as illustrated in the summary scheme in Fig. 5. The expression and methylation patterns of the glioma subtypes are shaped by the underlying key genetic defects in agreement with recent classifications of LGG [8, 45, 56]. Overall, we identified three consensus subtypes C1-C3 that were assigned as *IDH*-wt and *IDH*-mut astrocytoma-like and oligodendroglioma-like phenotypes according to their dominating genetic status in terms of the *IDH* mutation and Chr. 1p/19q codeletion. These genetic aberrations are assumed to act as early events of tumorigenesis [64] (see left part of Fig. 5a). A fourth, neuronal subtype (C4) collects specimen with reduced tumor cell content and served as reference partly resembling characteristics of healthy brain. However, our subtypes reflect also a large variability of expression and methylation phenotypes that do not match the genetic hallmarks in a one-to-one fashion. For example, 25–40% of all *IDH*-mut and 1p/19q-codel tumors were not assigned to the oligodendroglioma-like subtype (C3) but rather resemble the astrocytoma-like (C2) or neuronal (C4) types by a series of features. This heterogeneity results, among other possible factors, from the multidimensional nature of the transcriptomes and methylomes of the tumors. Each of their expression and methylation landscapes can be interpreted as a superposition of different expression and methylation patterns, which associate with specific cellular and micro-environmental states, and which obviously lack a clear-cut relation with respect to the underlying genotypes. The astrocytoma like gliomas constitute the largest fraction of nearly 60% of all LGG studied. They revealed the largest diversity and were divided into four expression (E2-E5) and three methylation (M2-M4) subtypes, which only partly match each other, thus reflecting partly decoupled expression and methylation patterns due to different possible interaction mechanisms [26, 57]. Particularly, decoupling between transcription and methylation can be rationalized in terms of independent regulation mechanisms of transcription by epigenetic and transcription factor (TF)-networks which are governed by bistable epigenetic switches [57]. Applying this model to cell differentiation data we recently identified situations where variant transcription of genes is accompanied by invariant epigenetic promoter states or vice versa. Interestingly, the former situation of TF-dominated regulation seems to apply to elementary cell functions related to stress response, cell cycle regulation and cell metabolism and requires mostly high expression levels of the genes beyond the sensitivity range of the switches. Combined regulation is found for developmental processes where genes become activated or deactivated by epigenetics, usually via histone methylation changes associated with DNA-hypo- or –hypermethylation near their promoters. Changes of methylation with only minor effect on transcription was found for *GPCRs* also upon cell differentiation. Overall we find striking agreement between gene functions in these three regimes between cell developmental data [57], WHO grade IV GBM [26] and the LGG studied here. Interestingly, methylation seems to-activate enhancers in TF-networks while it de-activates enhancers for developmental processes [72] or, in other words, enhancer and promoter methylation seem to act in an antagonistic fashion for both types of processes. A more simplistic interpretation of partly decoupled expression and methylation assumes rarely or non-overlapping sets of ‘passenger’ genes regulated by TFs and/or epigenetic ‘drivers’ such as the IDH-mutation [26].

On a cellular level, our results support a multi-component approach underpinned by single-cell transcriptome characteristics [59] that indicates variable composition of the tumors regarding healthy astrocyte- and oligodendrocyte-like cells, microglia/macrophage and proliferative stem cell-like constituents as illustrated in the right part of Fig. 5a. The TME of the subtypes is characterized by marked variations of the immune cell composition that overlays with methylation changes of their genomes. It suggests an epigenetically-mediated interplay between tumor cells and immune cells in the TME.

We found footprints of previously published expression and methylation gene signatures extracted from studies on WHO grade II, III and IV gliomas in the tumors studied here indicating a considerable overlap of molecular mechanisms between LGG and GBM [8] in agreement with previous studies which underlined relevance of GBM molecular signatures for LGG [21]. These results support the view that the molecular heterogeneity of gliomas decomposes into a set of gene-regulatory modes that were activated in different combinations and to a varying extent in the individual subtypes and in tumors of different grades. In addition to the G-CIMP and G-CIMP-O signatures that typically occur in *IDH*-mut gliomas we also found methylation characteristics occurring in *IDH*-wt GBM such as the RTK II and mesenchymal methylation signatures reflecting concerted methylation changes of respective groups of genes in *IDH*-mut LGG as well (Fig. 5a, part below). Moreover, we found concerted methylation patterns of the olfactory subgenome collecting *GPCR* genes and of cancer-related keratin intermediate filament genes, respectively. These signatures overlap and combine in different ways giving rise to diverse methylation and expression patterns that partly shape the glioma phenotypes.

### Phenotypic relatedness suggests developmental paths of gliomas

For a more detailed view on the relatedness between the subtypes we performed similarity tree analysis of the molecular tumor landscapes (Fig. 5b). The expression and methylation ‘phenotypic’ trees obtained differ mainly in the position of the *IDH*-wt (C1) subtype. Its expression characteristics show rather similarities with the C2 tumors because of common inflammatory and astrocytic signatures while its methylation profiles rather resemble that of neuronal (C4) tumors owing to the common lack of the G-CIMP patterns (see the schematic profiles in the lower part of Fig. 5b). On the other hand, both trees reflect similar mutual relations between the neuronal, oligodendroglioma-like (C3) and astrocytoma-like (C2) tumors where the former two types share similarities mainly regarding (low) inflammatory, (high) neuronal expression and (high) *GPCR*-methylation levels. Degeneration of apparent healthy brain functions in all subtypes, activated proliferation in C3 and partly inflammation in E3 seem to be driven by anti-correlated DNA-promoter methylation changes.

Interestingly, the astrocytoma-like subtypes in C2 sort in the order E4-E2-E3 and M4-M3-M2, respectively, which associates with increasing WHO grade of the tumors, their age at first diagnosis, their hazard ratio, the decrease of the global methylation levels and of neuronal expression characteristics and increased senescence bypass characteristics. We hypothesize that these trends reflect aspects of the progression of astrocytoma like gliomas from earlier to later phases in the natural course of the disease [2, 8]. Interestingly, these trends also suggest increasing therapy resistance along the proneural-to-mesenchymal axis after comparison with resistance and inflammatory signatures derived from GBM [54]. Search for glioma subtypes and/or molecular characteristics most suitable for immunotherapies is a challenge [43]. The inflammatory subtype E3 with maximum M2-macrophage polarization could be of interest for therapies targeting glioma associated macrophages [10].

Importantly, decreasing total methylation decomposes into reduced methylation of the *GPCR*- and keratin-methylation patterns on one hand, and the G-CIMP pattern, which shows the opposite trend in M4–M3, on the other hand (Fig. 5b, part below). The relative reduction of G-CIMP in M2 is compatible with the observation that while IDH-mut associated G-CIMP initiates gliomagenesis it seems not required for later clonal expansions [39]. Interestingly, the RTKII-signature originally obtained from WHO grade IV *IDH*-wild type gliomas shows parallels with the senescence bypass signature in *IDH*-mutated LGG, and particularly reflects differences between Chr1p/19q-codeleted and –non-codeleted tumors. Both, loss and gain of methylation take place in parallel in different regions of the genome of tumor cells and/or in different cellular constituents of the TME. The subtype E5 manifests characteristics of early stages of astrocytoma-like tumors such as high levels of the *GPCR*- and keratin-methylation patterns and low levels of the G-CIMP-methylation in addition to the expression of cancer testis genes. E5 collects both, Chr1p/19q-non-codeleted (mainly grade II) and, to a less amount, Chr1p/19q-codel tumors, which suggests that mechanisms affecting DNA methylation act partly independent of the Chr1p/19q-codel status.

### Aberrant methylation shapes glioma phenotypes

Gliomas are consistently characterized by the loss of neuronal expression signature, especially in *IDH*-wt and *IDH*-mut astrocytoma-like, and to a less degree, also in oligodendroglioma-like tumors, paralleled by decreasing expression and hyper-methylation of *PRC2*-targets with possible consequences for senescence bypass mechanisms. The latter properties are hallmarks of CIMP-like subtypes observed in colon cancer and lymphoma and in grade IV glioma [27, 38]. Previous studies have proposed a role for *PRC2* genes in protecting neurons against degeneration by repressing aberrant transcriptional programs [61]. Stratification of repressed chromatin states in fetal and adult brain revealed an antagonistic methylation pattern between oligodendroglioma-like (C3) and *IDH*-wt gliomas and an intermediate pattern in astrocytoma-like (C2) tumors which suggests deregulation of developmental cellular programs in *IDH*-wt; and of programs of differentiated tissue in C3 and partly also in C2. The former effect associates with the activation of inflammation and mesenchymal characteristics while the second one seems to activate proliferation and oxphos metabolism. Epigenetic activation of otherwise suppressed cellular programs seems to be essential for glioma development and diversification into subtypes.

Our methylation analysis uses integral methylation beta-values of upstream regions of each gene which are assumed to reflect mean promoter methylation levels. Similar methylation patterns were found in extended upstream regions, in the gene body and also for CpG-related signatures, which all together suggests that DNA-methylation affects widespread genomic regions in a similar fashion. On the other hand, this integral methylation analysis eventually overlooks local and CpG-specific methylation effects with possible impact for transcriptional regulation. In this context, our integral method should be judged as one limitation of this study. We expect that alternative methods will further improve our understanding of the role of DNA-methylation, e.g., to better resolve the regulatory element landscapes and transcription factor networks [72] in gliomas. Also the possible impact of methylation of the olfactory subgenome on cell function and glioma development remains partly unclear and requires future work.

## Conclusions

Our study demonstrates the importance of molecular subtyping of LGG as a concept to better understand the biology of this disease. We hereby follow a holistic approach which is guided by previous findings that diffuse gliomas can be further divided into epigenomic subtypes that differ in their biology with impact for treatment and prognosis beyond the WHO classification and histopathological grade. IDH mutated astrocytoma-like LGGs constitute the most heterogeneous sub-entity, which stratifies into distinct transcriptomic and methylation subtypes with possible impact for clinics, e.g. for identification of treatment resistant or sensitive tumor strata. Analogies between astrocytoma-like LGG with grade IV *IDH*-wt tumors regarding varying treatment resistance suggest similar disease mechanisms; however further studies are required for verification. Hereby epigenetics, and particularly, DNA methylation is a shaping and driving factor of glioma heterogeneity and progression. Genomic, epigenetic and transcriptomic factors act in concert but partly also in a decoupled fashion what underpins the need for integrative, multidimensional subtyping of LGG by combining these data on gene and cellular levels in order to delineate mechanisms of gene (de-)regulation and to enable better patient stratification for individualization of treatment.

## Additional files

Additional file 1: **Tables S3–S6.** Contains all supplementary figures and supplementary tables. (PDF 2870 kb)

Additional file 2: **Table S1.** List of GGN-patients whose DNA methylation data were included into the study. (XLSX 12 kb)

Additional file 3: **Table S2.** List of TCGA-samples used for verification analyses. (XLSX 18 kb)

## Abbreviations

2HG: 2-hydroxyglutarate
BP: Biological process
C1 – C4: Consensus classes of LGG with mutual correspondence between expression and methylation
CL: Classical subtype of grade IV gliomas according to (Verhaak, et al., 2010)
CN: Copy number
E1 – E8: Expression subtypes of LGG
EMT: Epithelial-mesenchymal transition
EPL: Early-progenitor-like subtype
G-CIMP: Glioma CpG-Island hyperMethylation Phenotype
G-CIMP-O: G-CIMP with specific hypermethylation of *IDH*-O
G-CIMP-wt: Hypermethylation of *IDH*-wt, particularly of the RTK II type
GEO: Gene expression omnibus database
GGN: German Glioma Network
GPCR: G-protein coupled receptor
GSZ: Gene set Z-score
HM: Hallmarks of Cancer
HR: Hazard ratio
IDH: Isocitrate dehydrogenase
IDH-A: Malignant astrocytoma-like
IDH-mut non-codel: *IDH* mutant with euploid 1p/19q
IDH-mut-codel: *IDH* mutant additionally carrying codeletion of chromosome arm 1p and 19q
IDH-O: Malignant oligodendrocytoma-like
IDH-wt: *IDH* wild-type
LGG: Lower grade gliomas (WHO grade II and III)
M1 – M6: Methylation subtypes
ME: Mesenchymal subtype of grade IV gliomas according to (Verhaak, et al., 2010)
MGMT: O-6-methylguanine-DNA methyltransferase
MP: Methylation pattern
MS: Mesenchymal subtype (Sturm et al. (reanalyzed by Hopp et al.))
NL: Neural subtype of grade IV gliomas according to (Verhaak, et al., 2010)
NPC: Neural progenitor cells
OS HR: Overall survival hazard ratio
Ox: Oxidative phosphorylation
PG: Pre-glioblastoma subtype of LGG (Gorovets, et al., 2012)
PN: Proneural subtype of grade IV gliomas according to (Verhaak, et al., 2010)
PRC2: Polycomb repressive complex 2
RTK: Receptor tyrosine kinase
RTKI: GBM methylation class (Sturm et al., 2012)
RTKII: GBM methylation class (Sturm et al., 2012)
SOM: Self-organizing map
TF: Transcription factors
TME: Tumor microenvironment
