# Supplementary material for: DNA methylation, transcriptome and genetic copy number signatures of diffuse cerebral WHO grade II/III gliomas resolve cancer heterogeneity and development
Source: Acta Neuropathol Commun. 2019 Apr 25;7:59. doi: 10.1186/s40478-019-0704-8 (PMC6482573; doi:10.1186/s40478-019-0704-8)
Supplement: Supplementary file 1 — Tables S3–S6. Contains all supplementary figures and supplementary tables. (PDF 2870 kb) [file 40478_2019_704_MOESM1_ESM.pdf]

Supplementary Text

DNA methylation, transcriptome and genetic copy number signatures of diffuse cerebral WHO grade II/III gliomas resolve cancer heterogeneity and development

H. Binder[#§1], E. Willscher[§1], H. Loeffler-Wirth[1], L. Hopp[1], D. T. W. Jones[2,3], S. M. Pfister[2,4,5], M. Kreuz[6], D. Gramatzki[7], E. Fortenbacher[1], B. Hentschel[6], M. Tatagiba[8], U. Herrlinger[9], H. Vatter[9], J. Matschke[10], M. Westphal[11], D. Krex[12], G. Schackert[12], J.C. Tonn[13], U. Schlegel[14], H.-J. Steiger[15], W. Wick[16,17], R. G. Weber[18], M. Weller[+7], M. Loeffler[+6]

1 Interdisciplinary Centre for Bioinformatics, Universität Leipzig, Härtelstr. 16–18, 04107 Leipzig, Germany

2 Hopp Children's Cancer Center Heidelberg (KiTZ), Im Neuenheimer Feld 430, 69120 Heidelberg, Germany

3 Pediatric Glioma Research Group, German Cancer Research Center (DKFZ), Im Neuenheimer Feld 280, 69120 Heidelberg, Germany

4 Division of Pediatric Neurooncology, German Cancer Consortium (DKTK), German Cancer Research Center (DKFZ), Im Neuenheimer Feld 280, 69120 Heidelberg, Germany

5 Department of Pediatric Oncology, Hematology and Immunology, Heidelberg University Hospital, Im Neuenheimer Feld 430, 69120 Heidelberg, Germany

6 Institute for Medical Informatics, Statistics and Epidemiology, University of Leipzig, Härtelstraße 16-18, 04107 Leipzig, Germany

7 Department of Neurology, University Hospital and University Zurich, Frauenklinikstrasse 26, 8091 Zurich, Switzerland

8 Clinic for Neurosurgery, Tübingen University Hospital, Hoppe-Seyler-Straße 3, 72076 Tübingen, Germany

9 Division of Clinical Neurooncology, Department of Neurology, University Hospital Bonn, Bonn, Germany

10 Institute of Neuropathologie, University Clinic Hamburg-Eppendorf, Martinistraße 52, 20246 Hamburg, Germany

11 Department of Neurosurgery, University Clinic Hamburg-Eppendorf, Martinistraße 52, 20246 Hamburg, Germany

12 Department of Neurosurgery, Technical University Dresden, Fetscherstraße 74, 01307 Dresden, Germany

13 Department of Neurosurgery, Ludwig Maximilians University Munich and German Cancer Consortium (DKTK), partner site Munich, Marchioninistraße 15, D-81377 Munich, Germany

14 Department of Neurology, University Hospital Knappschaftskrankenhaus Bochum-Langendreer, In der Schornau 23-25, 44892 Bochum, Germany

15 Clinic for Neurosurgery, University Düsseldorf, Moorenstr. 5, 40225 Düsseldorf, Germany

16 Clinical Cooperation Unit Neurooncology, German Cancer Consortium (DKTK), German Cancer Research Center (DKFZ), Im Neuenheimer Feld 280, 69120 Heidelberg, Germany

17 Neurology Clinic and National Center for Tumor Diseases, University Hospital Heidelberg, Im Neuenheimer Feld 400, 69120 Heidelberg, Germany

18 Department of Human Genetics, Hannover Medical School, Carl-Neuberg-Str. 1, 30625 Hannover, Germany

Supplementary Figures

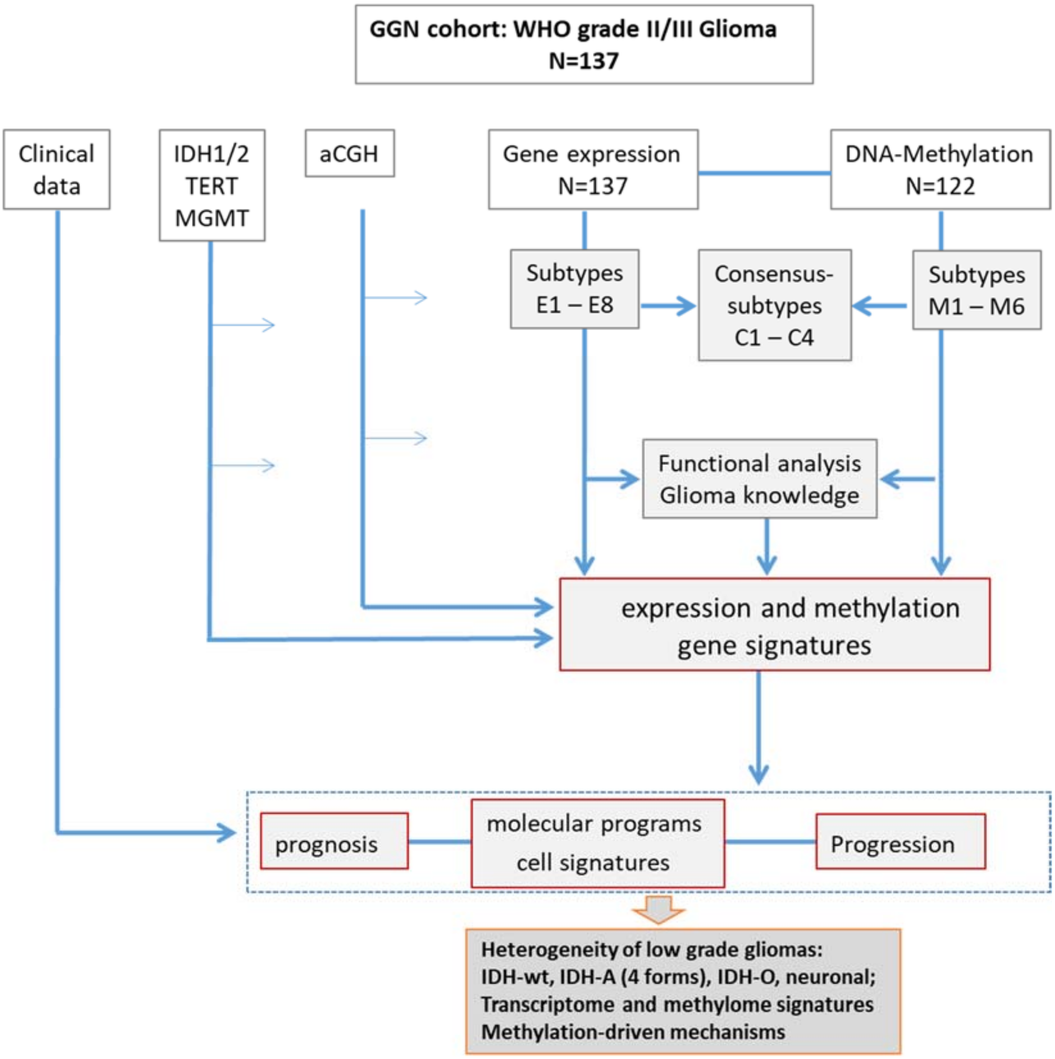

Figure S 1: Overview scheme summarizing data used, analyses and major objectives of the study

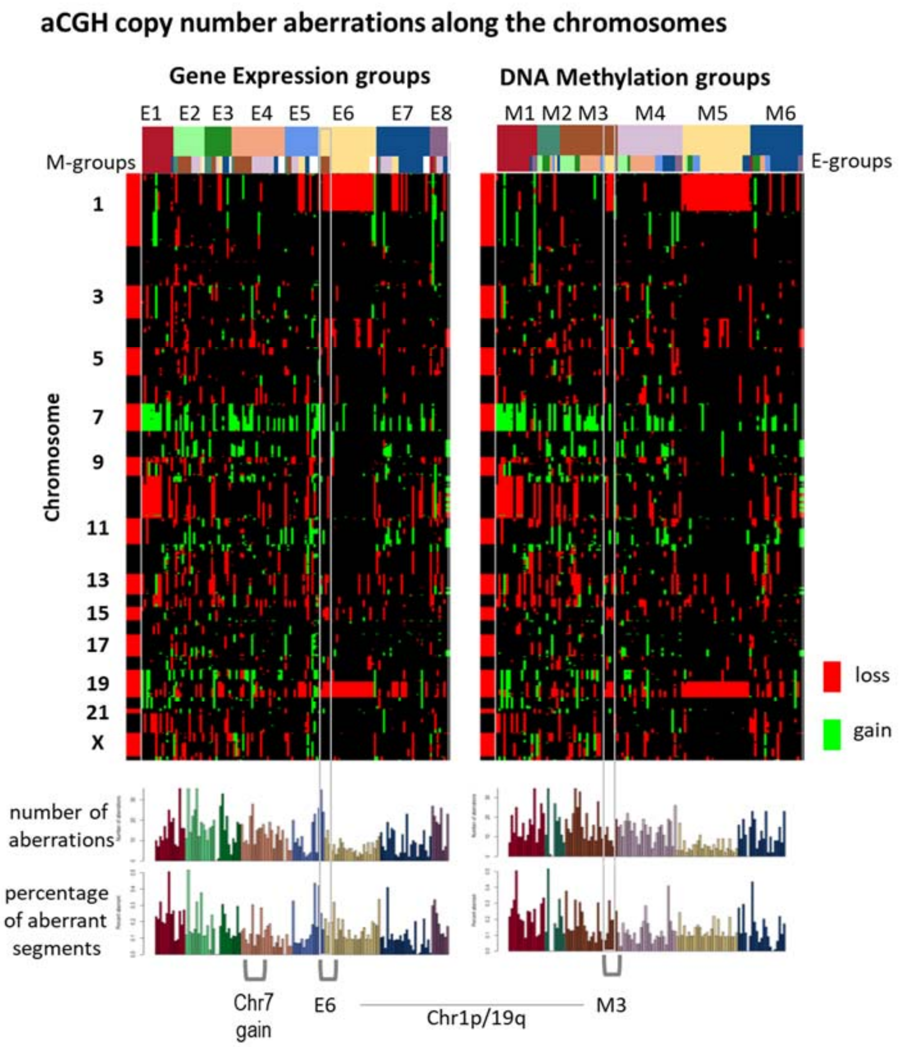

Figure S 2: Heatmap of copy number alterations in E- and M-groups of glioma samples. Copy number gains on Chr7 and losses on Chr10 accumulate in groups E1 and M1, whereas codeletions on Chr1p and Chr19q are frequently found in groups E6 and M5 which also are characterized by relative low total numbers of alterations. Copy number gains on Chr7 without Chr10 loss accumulate in samples of E4 and M3. Another combination is observed for E6 and M5 collecting samples with codeletions on Chr1p and Chr19q. Part of samples with these codeletions from E6 however sort into M3 (instead of M5, see the white frames in the figure) due to the different methylation of the olfactory subgenome (*GPCR*-signature) as indicated by the respective frames in Figure S 3.

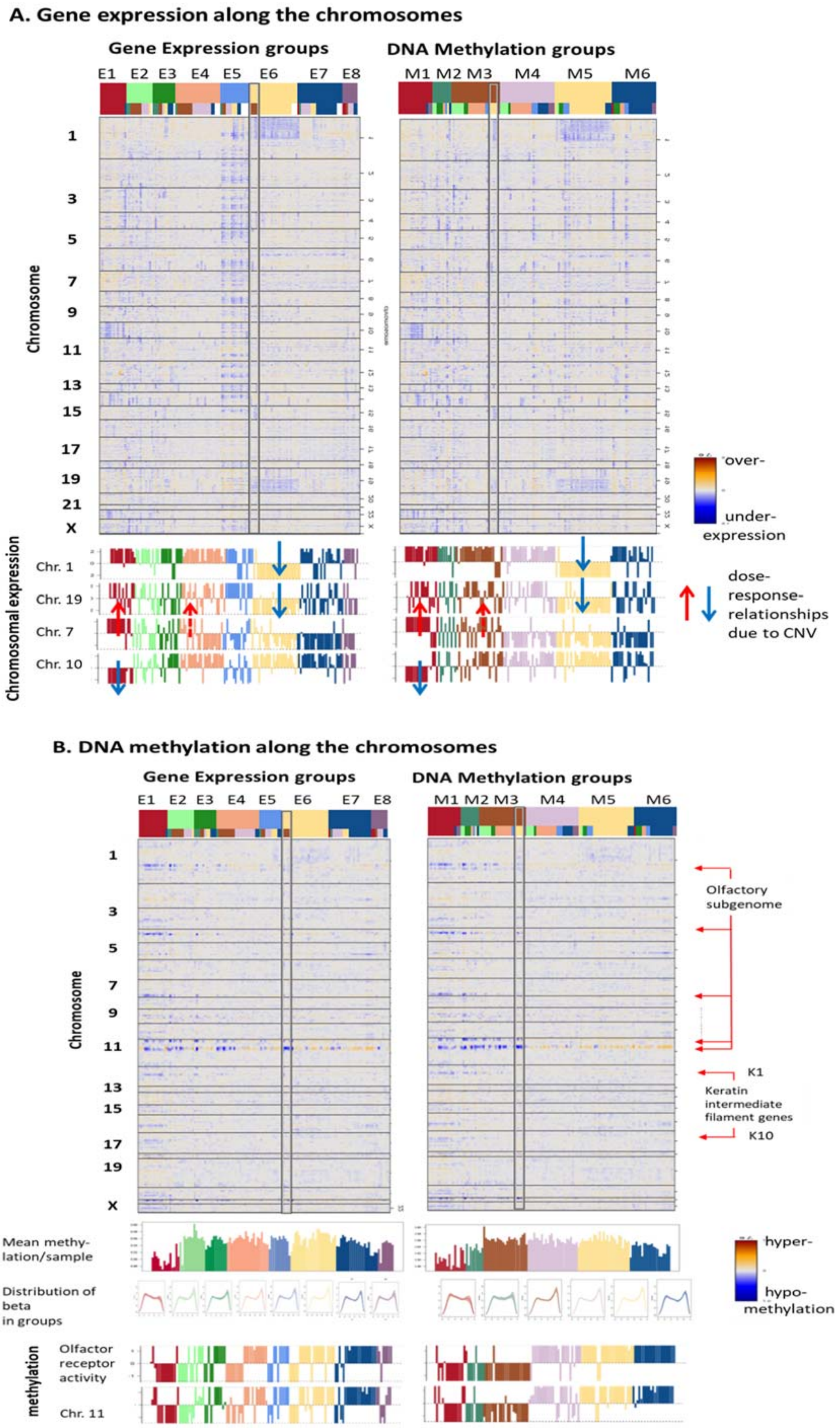
A. Gene expression along the chromosomes
Gene Expression groups
DNA Methylation groups
E1 E2 E3 E4 E5 E6 E7 E8
M1 M2 M3 M4 M5 M6
Chromosome
1
3
5
7
9
11
13
15
17
19
21
X
over-
under-
expression
Chromosomal expression
Chr. 1
Chr. 19
Chr. 7
Chr. 10
dose-
response-
relationships
due to CNV
B. DNA methylation along the chromosomes
Gene Expression groups
DNA Methylation groups
E1 E2 E3 E4 E5 E6 E7 E8
M1 M2 M3 M4 M5 M6
Chromosome
Olfactory
subgenome
K1
Keratin
intermediate
filament genes
K10
Mean methy-
lation/sample
Distribution of
beta
in groups
hyper-
hypo-
methylation
methylation
Olfactor
receptor
activity
Chr. 11

Figure S 3: Gene expression (part A) and methylation (part B) along the chromosomes was estimated using sliding-windows averaging [1] to study positioning effects of the genes. Chromosomal gene expression (part A) reveals regions of increased and decreased transcription due to copy number aberrations especially on Chr1, 7, 10 and 19. The mean expression of all genes from the respective chromosomes clearly indicates up- and downregulation for copy number gains and losses, respectively (part A below, see arrows). Positioning effects of methylation (part B) are evident in regions of the olfactory subgenome especially on Chr11 [2] and of the keratin intermediate filament gene clusters K1 and K10 [3]. The methylation profile of the gene set 'olfactory receptor activity' containing 175 genes coding olfactory receptors (75 of them on Chr11) closely agrees with the methylation profile of all genes from Chr. 11. The methylation profile of olfactory receptors reflects a sharp cut between methylation groups M1 to M3 (hypomethylated) and M4 to M6 (hypermethylated). The mean absolute methylation per sample is high for *IDH* mutated cases which accumulate in groups E2 to E5 and M2 to M5. The frequency distribution of beta values in general shows a bimodal shape with its maximum either at low or high beta for *IDH* wild type and mutated cases, respectively.

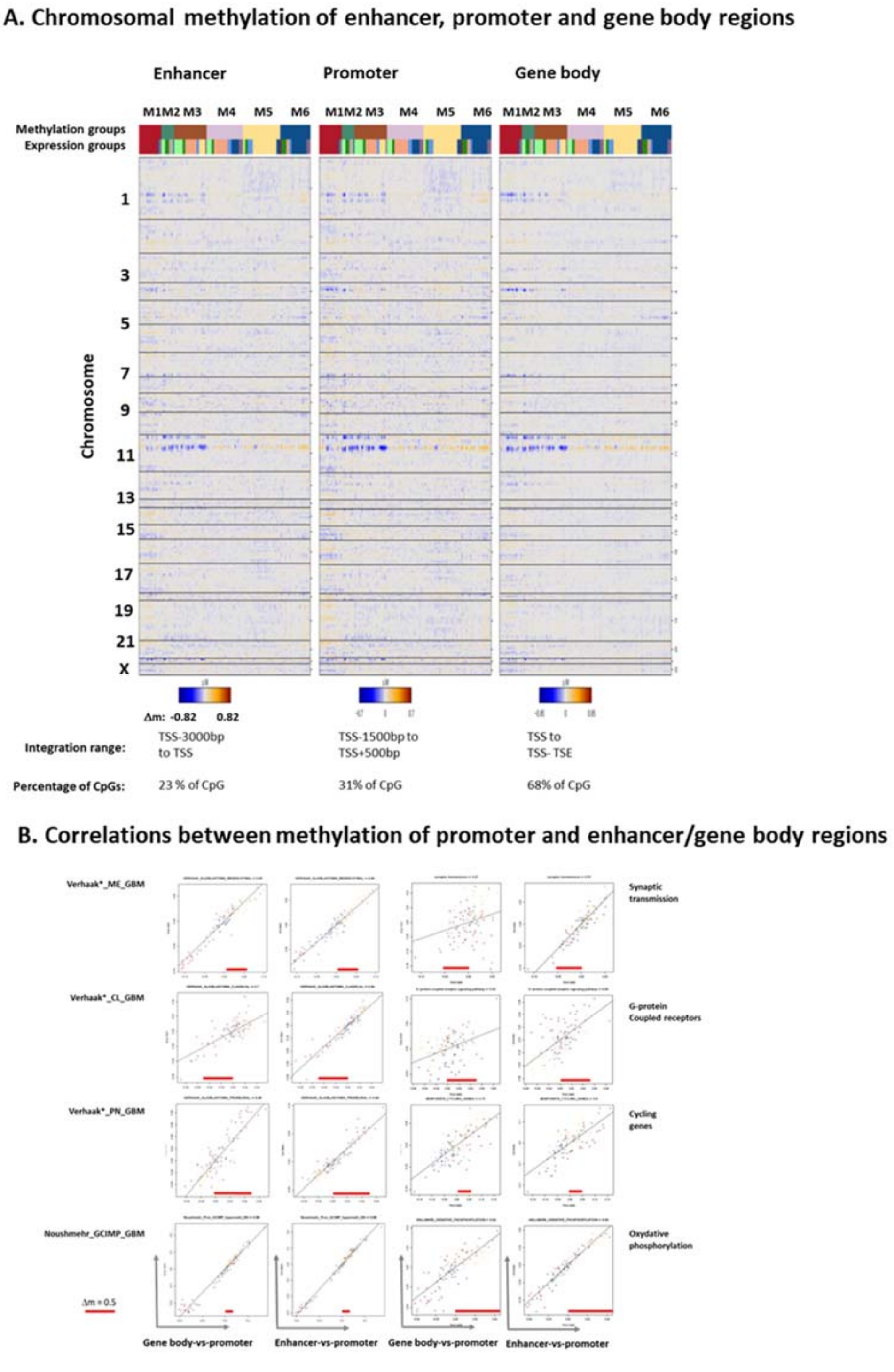

Figure S 4: The effect of DNA-methylation in gene enhancer, promoter and gene body regions: A) Methylation along the chromosomes is virtually identical on this rough scale. Particularly one finds hypomethylation of the olfactory subgenome in all three regions upstream and downstream of the TSS. B) Correlation plot between gene body/enhancer versus promoter regions for genes of different signature sets. For most of the signatures we find strong correlations (r^2>0.8) indicating similar mean methylation trends in enhancer, promoter and gene body regions. DNA methylation in the three different regions was calculated as the mean m-value (m= log [β/(1-β)]) averaged over CpG-probes over distinct genomic regions as indicated in part A of the figure (TSS…transcription start site; TSE…transcription end site). Overall these data suggest aberrant methylation in widespread regions of the genome. This notion is further supported by the result that marker CpGs randomly distribute over the three enhancer, promoter and gene body ranges in proportions 2:3:7 (Figure S 11).

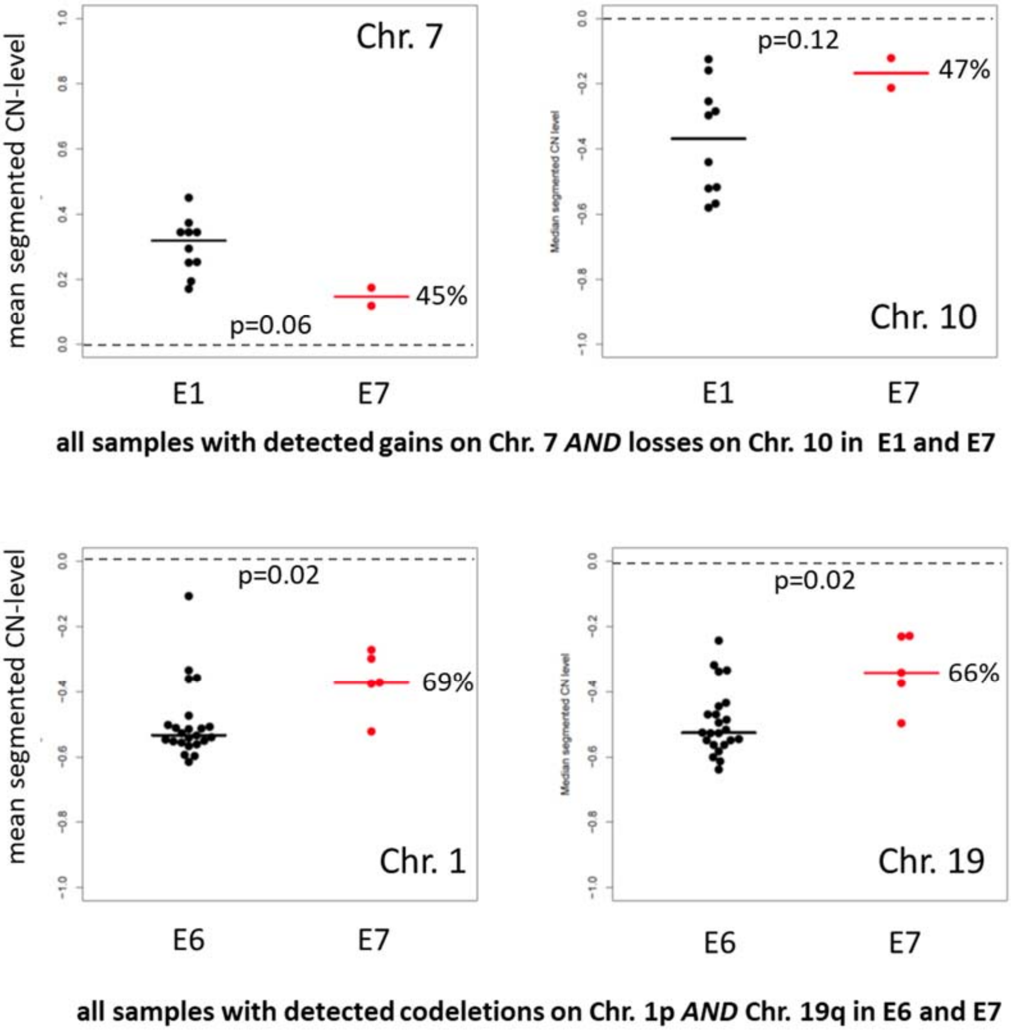

Figure S 5: Comparison of copy number (CN) levels on selected chromosomes between samples in subtype E7 and E1 showing gains and losses on Chr7 *and* Chr10, respectively, and between samples in subtypes E7 and E6 showing codeletions on Chr1p *and* Chr19q. The systematically smaller absolute levels of the CN in E7 compared with E1 and E6 suggests contaminations with healthy cells without these CN defects. Note that subtype E1 is strongly enriched in samples showing gains on Chr7 and losses on Chr10 (10 out of 14 samples) whereas subtype E6 strongly enriches in samples showing codeletions on Chr1p and Chr19q (24 out of 26 samples). On the other hand, subtype E7 (24 tumors in total) is a relatively heterogeneous mixture that contains samples showing either the one (2 samples with Chr7 gains and Chr10 losses) or the other (6 samples with Chr1p and Chr19q codeletions) key defect. The relative decrease of CN varies between (45-47)% (Chr7 gains and Chr10 losses) and (66-69)% (Chr1p and Chr19q codeletions) which roughly estimates the relative decrease of tumor cell content. p-values (t-test) for group differences were given in the figure. Note that expression analysis assigns gene signatures of healthy brain to E7 but not to E1 and E6 which supports the results of copy number analysis. Overall, these results support the view that E7 collects samples contaminated with healthy brain tissue.

A. Overall survival curves of groups

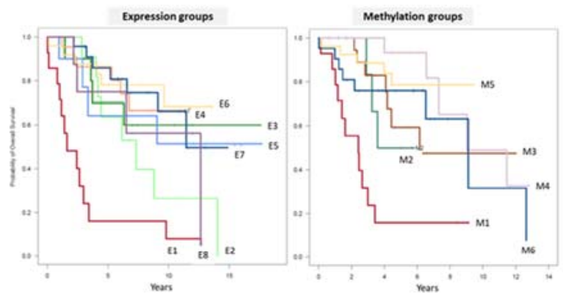

B. Overall survival curves of groups vs others

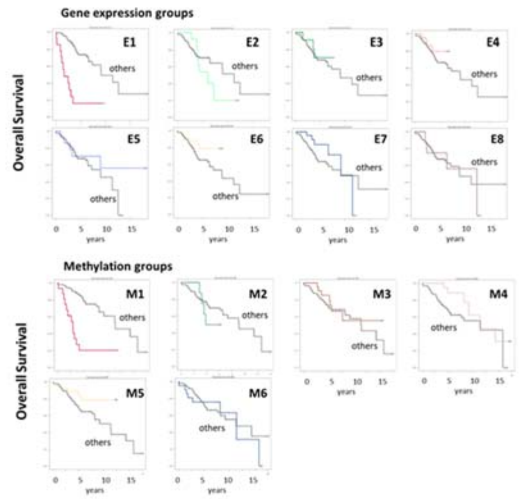

C. p-values of Hazard Ratio comparisons

group	E2	E3	E4	E5	E6	E7	E8
E1	9,00E-03	1,00E-03	2,90E-05	0.003	1,50E-05	1,80E-05	0.02
E2		0.4	0.41	0.28	0.03	0.13	0.4
E3			0.79	0.85	0.5	0.4	0.87
E4				0.5	0.88	0.5	0.75
E5					0.5	0.9	0.57
E6						0.8	0.48
E7			p<0.05				0.77
E8							

group	M2	M3	M4	M5	M6
M1	3,00E-02	7,00E-04	1,52E-05	4,00E-05	1,00E-02
M2		0,37	1,40E-02	0,1	0,4
M3			0,2	0,34	0,99
M4				0,86	0,18
M5					0,33
M6		p<0.05			

D. Overall survival Hazard Ratios

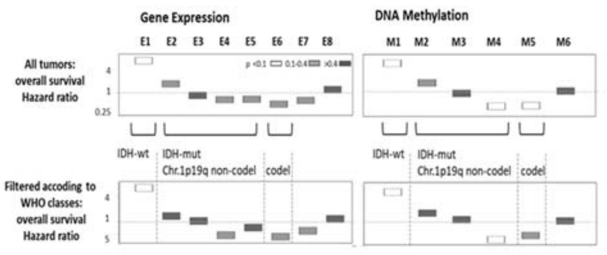

Figure S 6: Survival analysis of the E- and M-groups: A) Overall survival (OS) curves for E- and M-groups. Interestingly, the OS-curves of M-groups M3 – M6 seem to reflect more prognostic differences than the E-groups E3 – E8 which is supported also by the respective p-values (part C of the figure). However larger sample sizes are required for justification of this result. Note that the IDH-mut groups E2 (IDH-wt resemblance) and M2 (low GCIMP) reveal worst survival among the IDH-mut groups. B) OS compared with that of the remaining samples of the cohort (‘others’). C) Signifinace p-values of pairwise comparisons of hazard ratios (HR). E1 and M1 (IDH-wt) compared with the other groups (mostly IDH-mut) show significantly worse prognosis. E2 reveals unfavorable prognosis compared with the other E-groups. Similar prognosis applies to M2 among the M-groups. D) HRs of the groups were shown as boxplots. The HR data were calculated considering all tumors in each of the groups (upper row of figures) and after filtering the groups in accordance with WHO classifications (i.e., considering only IDH-wt in E1 and M1; IDH-mut and Chr.1p/19q non-codel in E2 – E5 and M2 – M4; IDH-mut and Chr.1p/19q codel in E6 and M5). The effect of filtering is small and doesn’t change the general trends discussed in the paper.

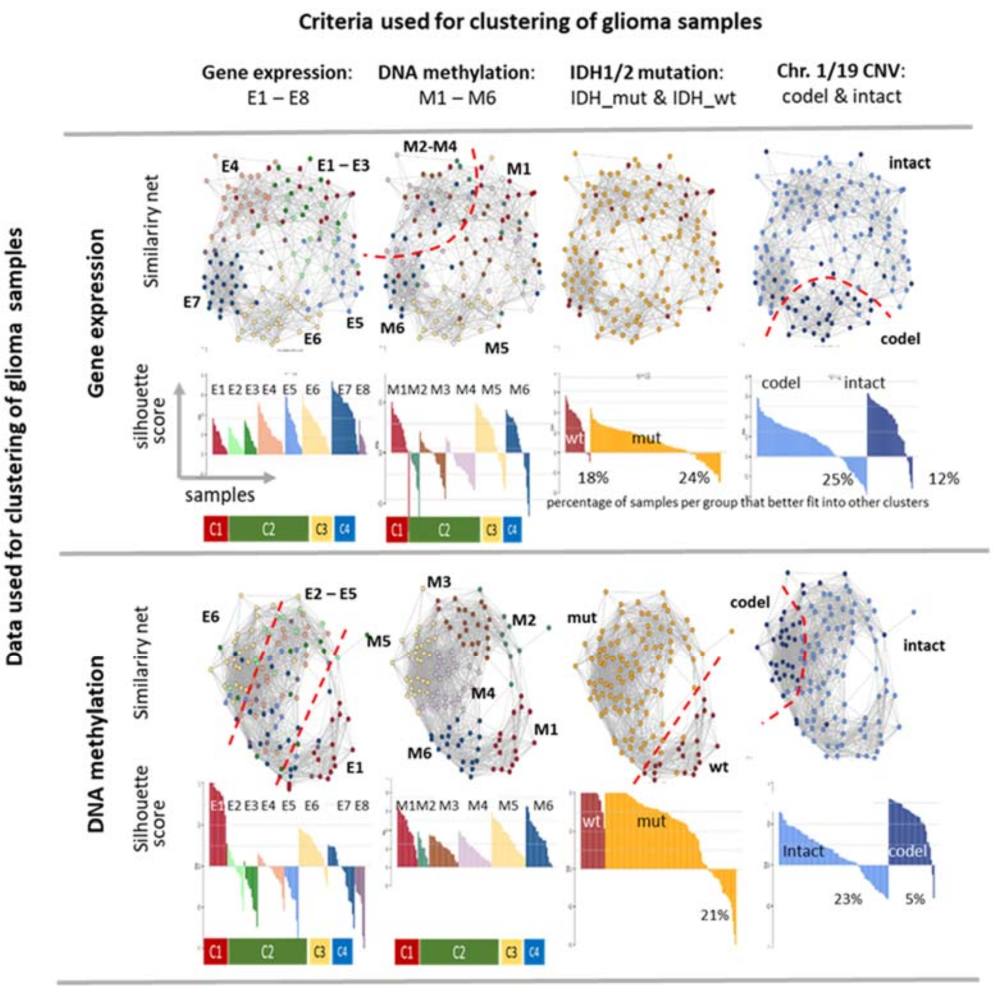

Figure S 7: Coloring of the samples according to different classification categories (see column headers) in the similarity network representation of the sample landscapes of expression (row above) and methylation (row below) data reveals different degrees of fuzziness of the class assignments: 1p/19q codeleted (and *IDH* mutated) tumors form clearly separate clusters in the methylation and expression landscapes as well. The *IDH* mutated and non-mutated tumors well separate each from another in the methylation landscape while their mutual distributions in the expression landscape are fuzzier. The samples of the expression subtypes E1 – E8 and that of the methylation subtypes M1 – M6 collect into almost severed clusters in the E- and M-nets, respectively. Here the assignment of samples to the subtypes meets the criterion of maximum silhouette scores used for classification. This score estimates the preference for each sample being member of the actual subtype as best choice compared with the second best choice as a member of another subtype using a correlation coefficient metrics [4]. The silhouette score estimates the optimal cluster membership of the samples with positive values indicating preference for the actual cluster chosen and negative values for alternative cluster memberships. It is always positive for the E- and M-subtypes using the expression and methylation data, respectively, thus indicating optimal clustering. Reversal of the labels, i.e. coloring of the samples in the E-net according to their M-label and vice versa shows still separate clouds for the samples subsumed in consensus classes C1, C3 and partly C4 but considerable mixing of the subtypes in C2 in agreement with the results presented in the main paper (Figure 1). The silhouette plots also show that typically about 20% of the samples with *IDH* mutations show closer similarities with the respective alternative clusters of non- *IDH* mutated tumors according to their expression and methylation patterns. Although the status of codeletions on Chr.1p/19q well separates the tumors in the E- and M-net as well about 5 – 25% of the samples of each group show closer similarity to the alternative group as indicated by the negative values of their silhouette score. Overall, this analysis shows that our subtyping shows clear associations between the gene expression and methylation landscapes and with the main genetic hallmarks. It is however overlaid by a certain degree of uncertainty reflecting intermixing of genetic, DNA methylation and transcriptional effects.

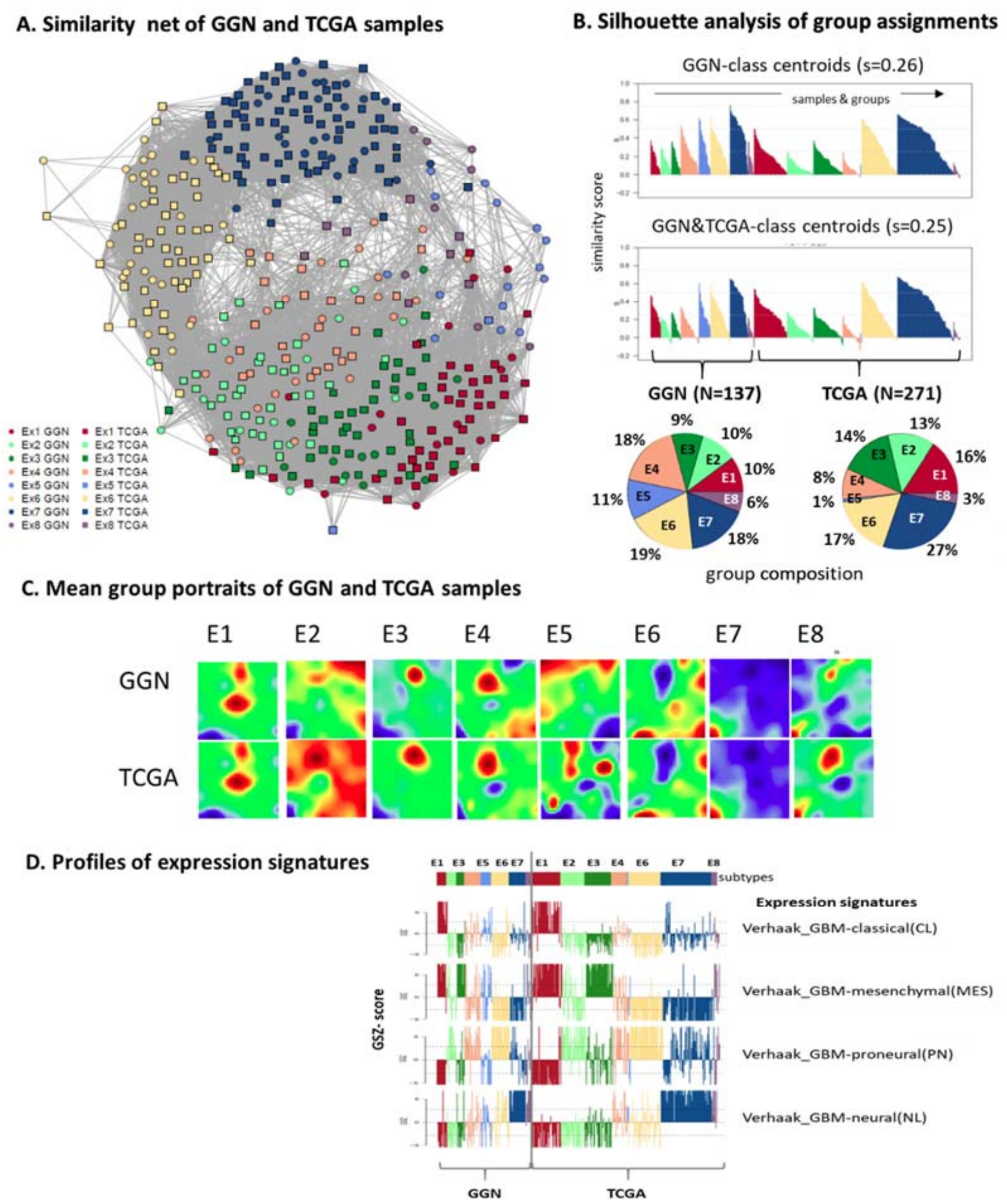

Figure S 8: Validation of the subtyping of the expression landscapes using a TCGA cohort of lower grade (II/III) glioma that comprises matched expression data of 271 tumors and methylation data of 278 tumors (Table S 2). Both, the GGN and TCGA data sets of LGG samples were trained together using a GGN-guided extension SOM-clustering of the TCGA samples [5]. TCGA samples were classified according to closest similarity to the class centroids of the GGN-samples. A) The similarity network confirms that GGN (circles) and TCGA (squares) samples of the same class cluster together and form common data clouds. B) Predominantly positive values of the silhouette cluster scores for GGN and TCGA samples confirm proper classification. Using cluster centroids of the GGN-samples of each class or of both, the GGN and TCGA samples, provided almost identical results. All sample groups except E5 were reproduced in the TCGA data. The compositions of both cohorts regarding the subtypes except E5 resemble each other. C) Mean SOM-group portraits of the GGN- and TCGA-cohorts confirm agreement of the expression landscapes of the subtypes except E5. Details and possible reasons for this fact will be discussed in the main paper. D) Expression profiles of transcriptional signatures of the Verhaak-GBM classes (see Table S 6) across the E-subtypes assigned to samples of the GGN and TCGA data sets. Both data sets are in strong agreement with subtype related expression. Note also the sharp changes of the expression levels from subtype-to-subtype which confirms proper classification. Interestingly, E3 shows mesenchymal and reduced proneural characteristics in contrast to the other IDH mutated subtypes what explains the partial similarity between E3 and the IDH-wt E1- groups evident in the heatmap in Figure 1A.

SOM extension method: The SOM extension method [5] aims at adding new, secondary sample data (e.g. TCGA tumors here) to an already existing SOM (e.g. that of the GGN tumors here) and thereby adapting the secondary data to the primary SOM space. This extended SOM is obtained by repeating the training process for the combined data where however only the primary data were used to calculate the intrinsic metrics of the SOM during initiation and adaptation. This 'pickaback' training assigns unchanged metagene values to the primary data and new, adapted metagene data to the secondary data.

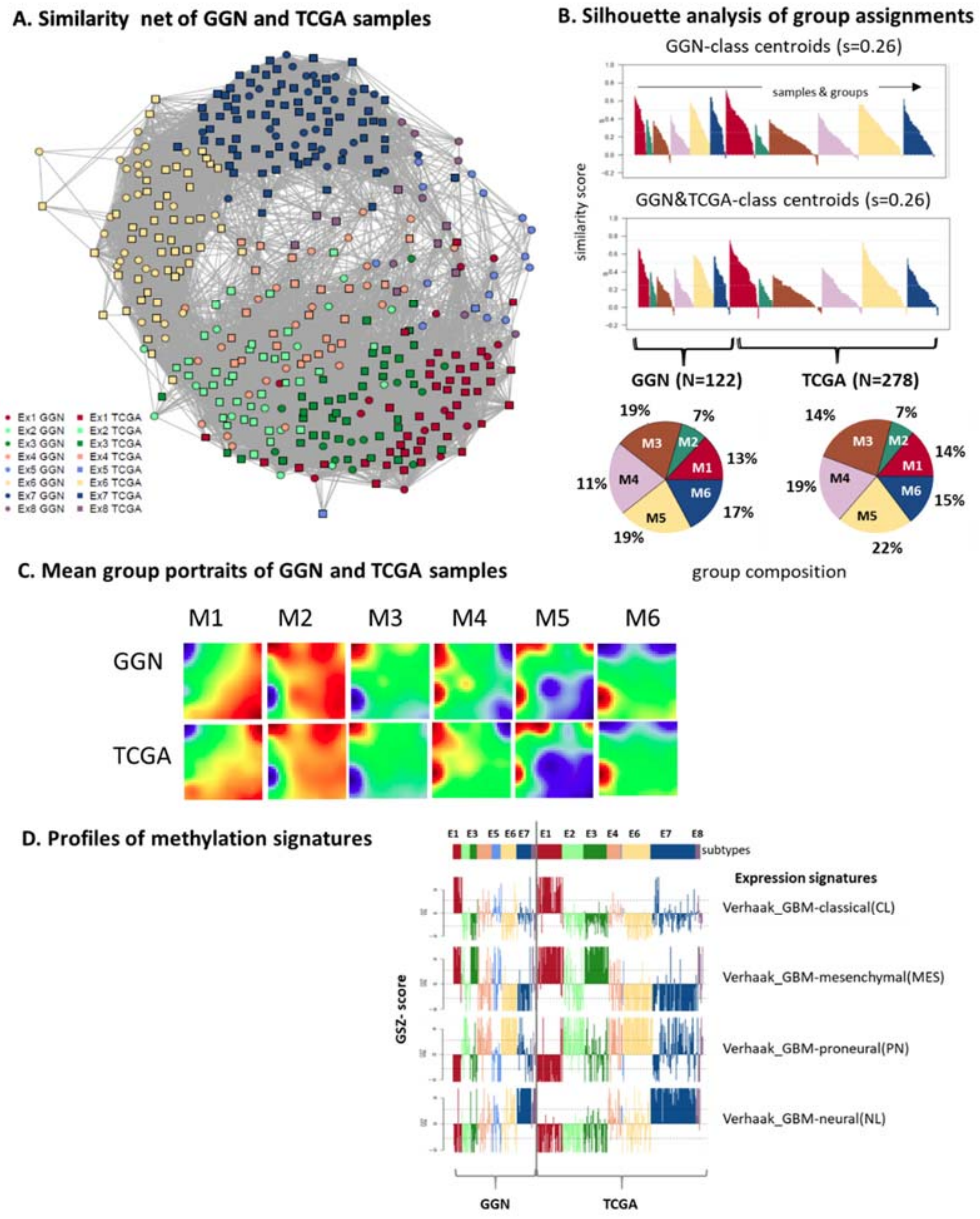

Figure S 9: Validation of the subtyping of the methylation landscapes using the TCGA cohort of lower grade glioma. All methylation subtypes were reproduced in the GGN cohort. See legend of Figure S 9. Also a set of marker CpG-genes published in [6] for classification of LGG well differentiates between the most of the M-groups identified here (Figure S 10). D) Methylation profiles of methylation signatures as proposed by Sturm et al. (Table S 6) and of *GPCRs* across the subtypes assigned to samples of the GGN and TCGA data sets. Both data sets are in strong agreement with subtype related methylation

patterns, e.g. of the IDH subtype reflecting hypermethylation in IDH-mut tumors of both data sets, but also for the GPCR signature showing hypomethylation in M1 – M3 in GGN and TCGA data as well. Note that the RTKII and MS methylation signatures designed to classify IDH-wt GBM differentiate between IDH-O and IDH-A LGG samples of the GGN and TCGA cohorts as well.

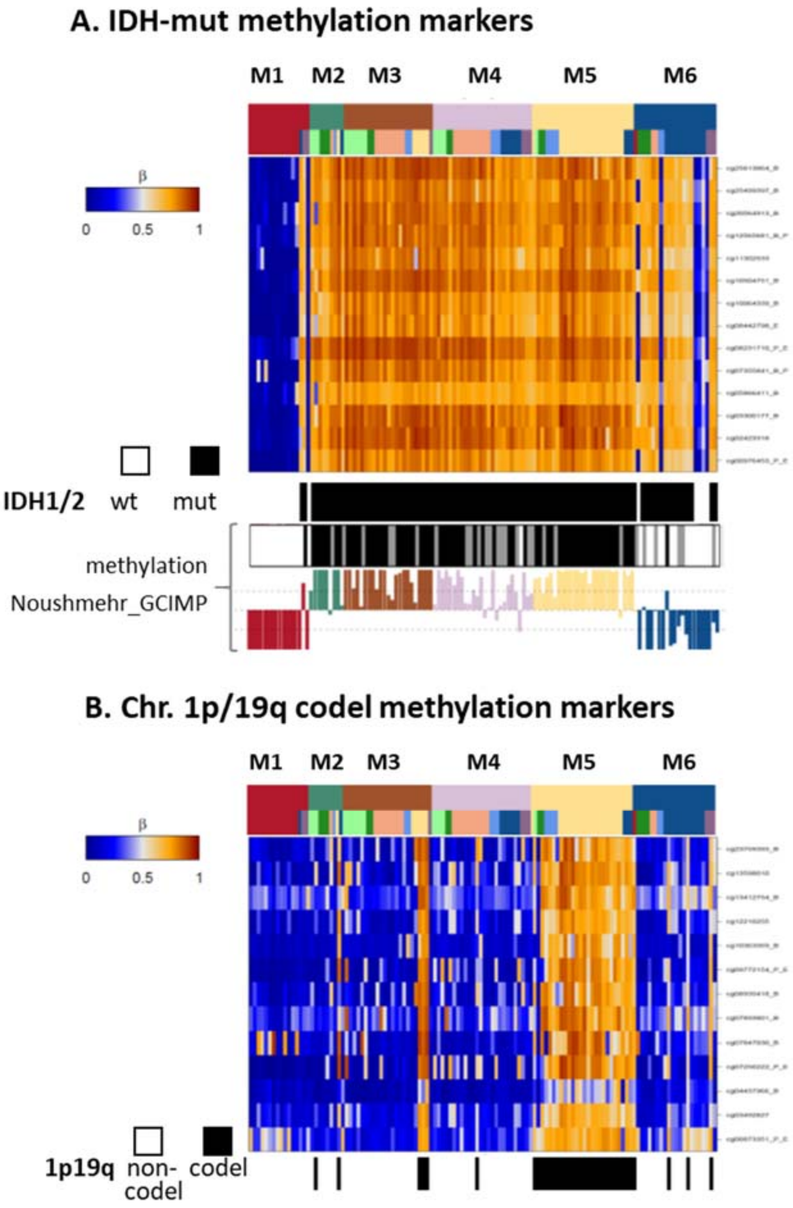

Figure S 10: Heatmaps of marker CpGs for IDH mutated (part A) and Chr 1p/19q codel (B) tumors taken from [7]. Both methylation profiles confirm the genetic data shown as black (mutated/aberrant)/white (non-mutated/intact) barcodes for IDH mutated and Chr 1p/19q tumors, respectively. Also the signature of Noushmehr et al. (Table S 6) which considers hypermethylated and underexpressed genes in GCIMP tumors agrees with the IDH-mut status however with less contrast in the M6 (neural) subtype presumably due to an relatively high content of healthy tissue in M6 samples.

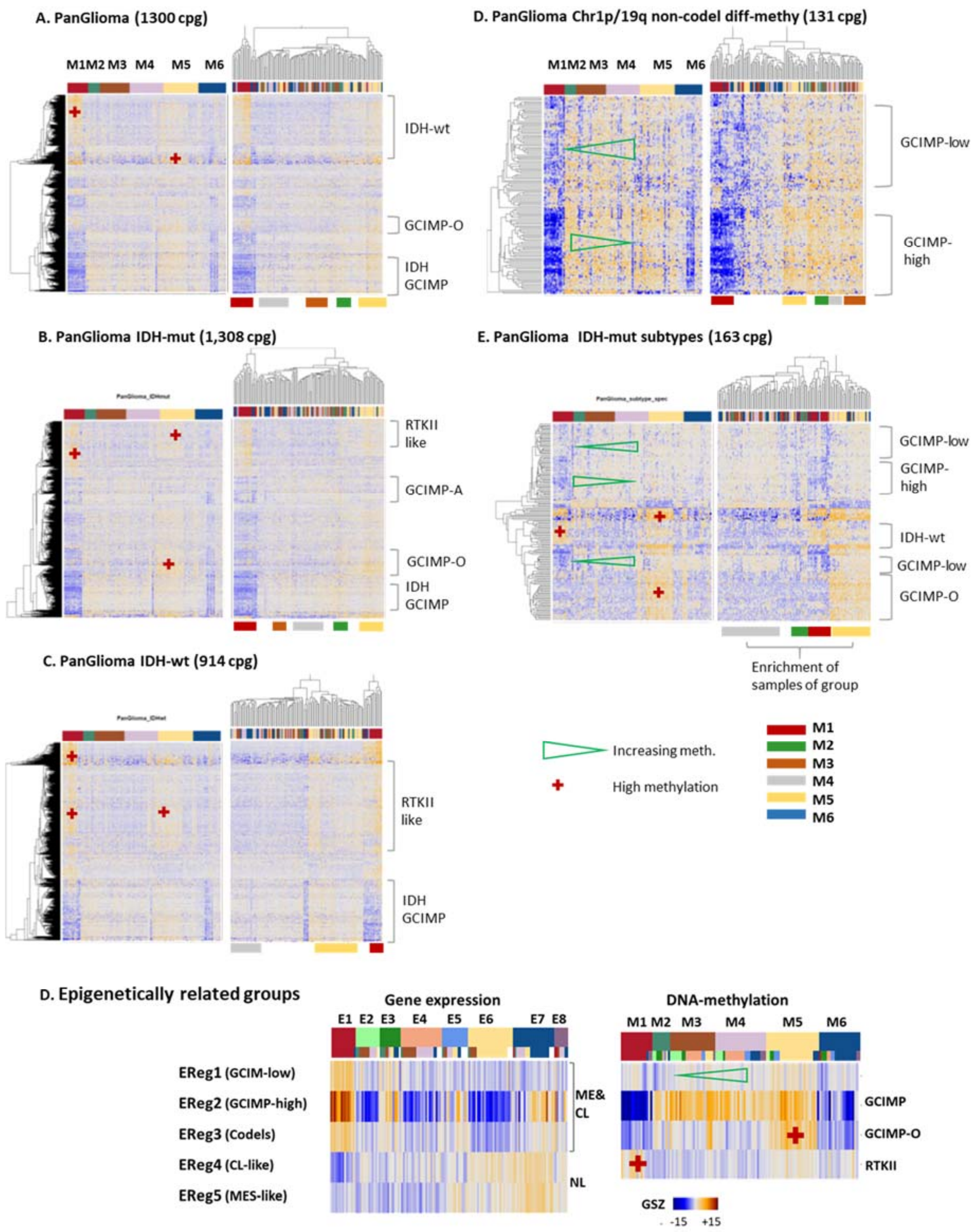

Figure S 11: CpG methylation and epigenetically regulated gene signatures of glioma subclasses taken from [6] (https://tcga- data.nci.nih.gov/docs/ publications/lgggbm_2015/) were applied to samples studied in this work. They were used in [6] for classifying gliomas into different subclasses. Particularly CpG signatures were used for clustering A) gliomas into 6 methylation (Lgm) classes; B) IDH-mut glioma into 3 (K1- K3) classes; C) IDH-wt gliomas into 3 (K1 – K3) classes; D) IDH-mut Chr1p/19 non-codel into two classes (GCIMP-high and GCIMP.low); E) IDH-mut gliomas into three classes (co-del, GCIMP-high, GCIMP-low). The left heatmap in each parts sorts the samples according to M-groups while the samples in the right parts are sorted using unsupervised hierarchical clustering. Overall, the signatures show structured methylation changes between most of our subtypes (and vice versa), especially IDH-

wt, IDH-O and also IDH-A were consistently reproduces (see also Figure S 12). Note that also M2 as a GCIMP-low like group is identified (part D and E). The CpG signatures reveal footprints of the signatures discussed in Figure 2. Overall they reveal specific hyper-methylation properties for M1 (IDH-wt), M5 (IDH-O) and differences of methylation between M2 (IDH-A, GCIMP-low like) and M3-M4 (IDH-A, GCIMP-high like) which reflects agreement with our data and that published in [6]. D) Epigenetically related groups (ERegs) were taken from [6] in terms of CpGs and related genes and mapped onto our E- (gene expression) and M- (CpG-methylation) groups and presented as GSZ-score for each group. EReg1 characterizing GCIMP-low tumors in [6] reveals and decreased methylation level in M2. EReg2 (GCIMP-high) resembles the IDH methylation and mesenchymal (ME) and partly classical (CL) expression profiles (compare with Figure 2). Analogies between ERegs 3 -5 and other previous signatures such as GCIMP-O and RTKII is evident after comparison with Figure 2. Overall comparison with EReg enabled us to assign our subtypes to that published in [6] (see below Figure S 13B).

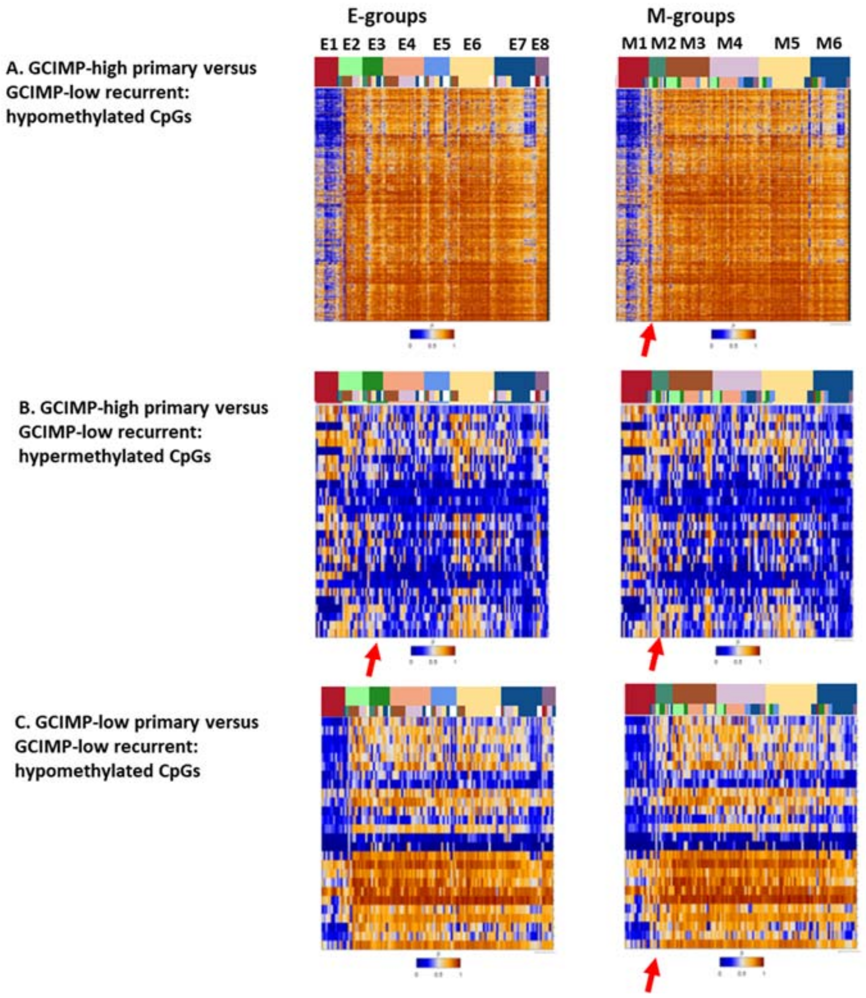

Figure S 12: CpG signatures characterizing glioma recurrence taken from [8] were mapped on GGN methylation data and sample sorting according to E- and M-groups. Differentially hypo-methylated (part A) and hyper-methylated (part B) CpG sites between G-CIMP-high primary tumors and their G-CIMP-low first recurrent counterparts. C) Differentially methylated CpG sites between G-CIMP-low primary tumors and G-CIMP-low first recurrent tumors. One finds that methylation changes in recurrent tumors is also observed in M2 and also E2 and E3 (see red arrows).

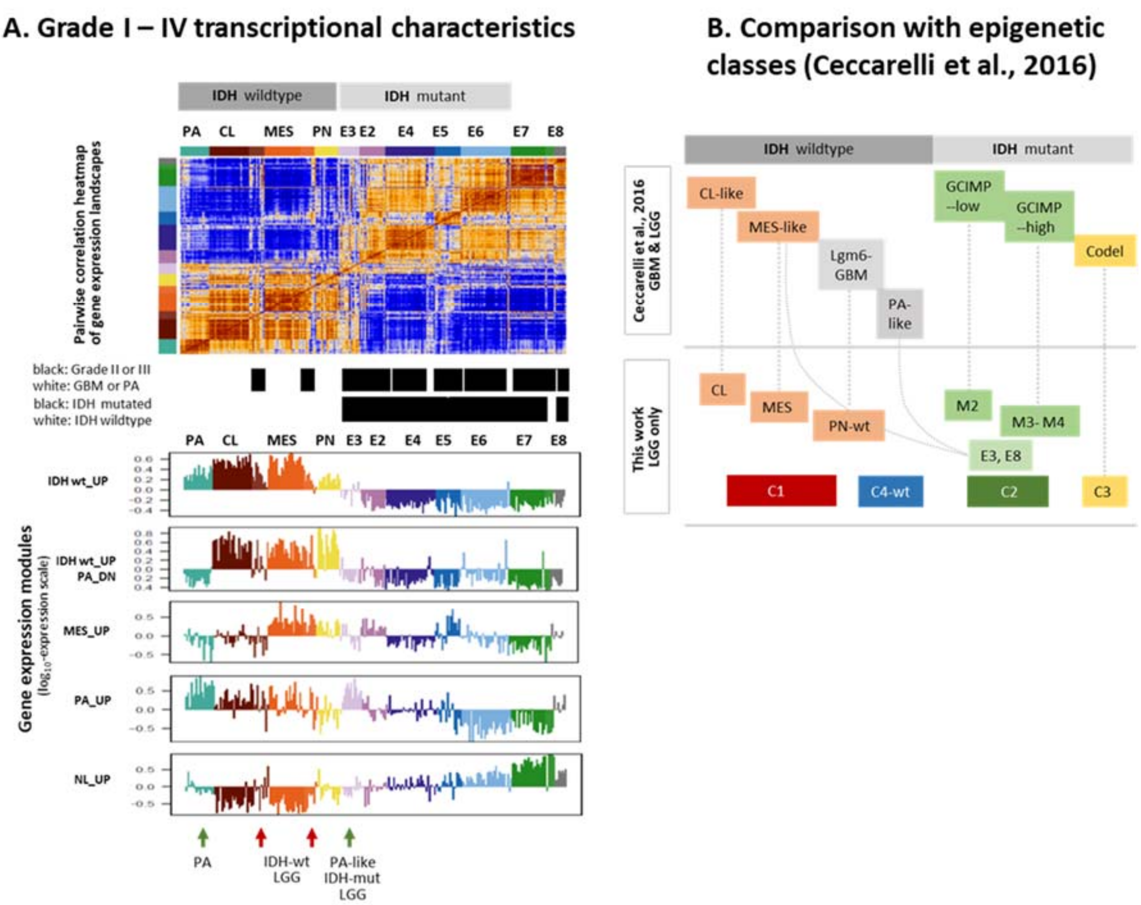

Figure S 13: Comparison of classification of LGG proposed in this work with the epigenetic classes of glioma provided by Ceccarelli and colleagues [6]. (A) Pairwise correlation heatmap of the full GGN-glioma data set, which comprises the LGG studied in this work, 94 grade IV GBM described previously [9] and 16 pilocytic astrocytomas (PA) collected in the GGN. Our LGG data set was extended by GBM and PA to make it comparable with the glioma set of Ceccarelli et al. For a joint subtyping which includes grade I to IV gliomas we classified all IDH-wt LGG to the GBM IDH-wt GBM subtypes used in [9], namely CL (classical), MES (mesenchymal) and PN-wt (proneural) except for the neural ones (E7 and E8). All twelve IDH-mut grade IV GBM were classified into the LGG classes E2 – E8 (see the class label bar and the WHO-grade bar in the figure). Then, the transcriptomic data were clustered into five major modules of co-regulated genes whose profiles were shown below the heatmap. Firstly, we find that expression of E3 resembles that of PA (green arrows) while expression of E2 partly resembles that of MES IDH-wt GBM. Secondly, grade II and III IDH-wt tumors assigned to CL and MES subtypes slightly differ from grade IV IDH-wt by elevated neuronal characteristics (NL_UP module) and reduced IDH-wt characteristics (see red arrows). Note also the continuous decay of the NL_UP signature from E7 to E3/E2 and IDH-wt which roughly associates with increased grading and/or tumorigenicity. (B) The scheme compares our subtypes with the seven classes of Ceccarelli et al. Our CL, MES and PN-wt tumors correspond to the CL-like, MES-like and Lgm6-GBM classes of Ceccarelli et al., respectively, where Lgm6 enriches MS (mesenchymal methylation signature) tumors which associates with neuronal C4-group tumors in our cohort (see Figure 2). The PA_UP profile in panel A suggests that part of the CL and especially MES tumors resemble PA-characteristics and eventually can be assigned to a PA-like subtype not explicitly considered by us. More interestingly, E3 and, partly, E8 IDH-mut LGG also show PA-resemblance. The GCIMP-low, GCIMP-high and codel classes of Ceccarelli et al. can be assigned to our classes M2 – M5 as indicated by the dashed lines. Note also that M2 shows worse prognosis compared with the other IDH-mut groups in correspondence with the poor prognosis of GCIMP-high tumors while E3 shows relatively good prognosis in correspondence with PA-like tumors of Ceccarelli et al. (see Figure S 6). Note also good agreement between M2 (9% of IDH-mut of grade II-IV in GGN) and GCIMP-low (6% of IDH-mut tumors of grade II-IV in TCGA) regarding the percentage of cases. Hence, our data support the classification of Ceccarrelli et al. while we, in addition, identified IDH-mut LGG with PA-resemblance.

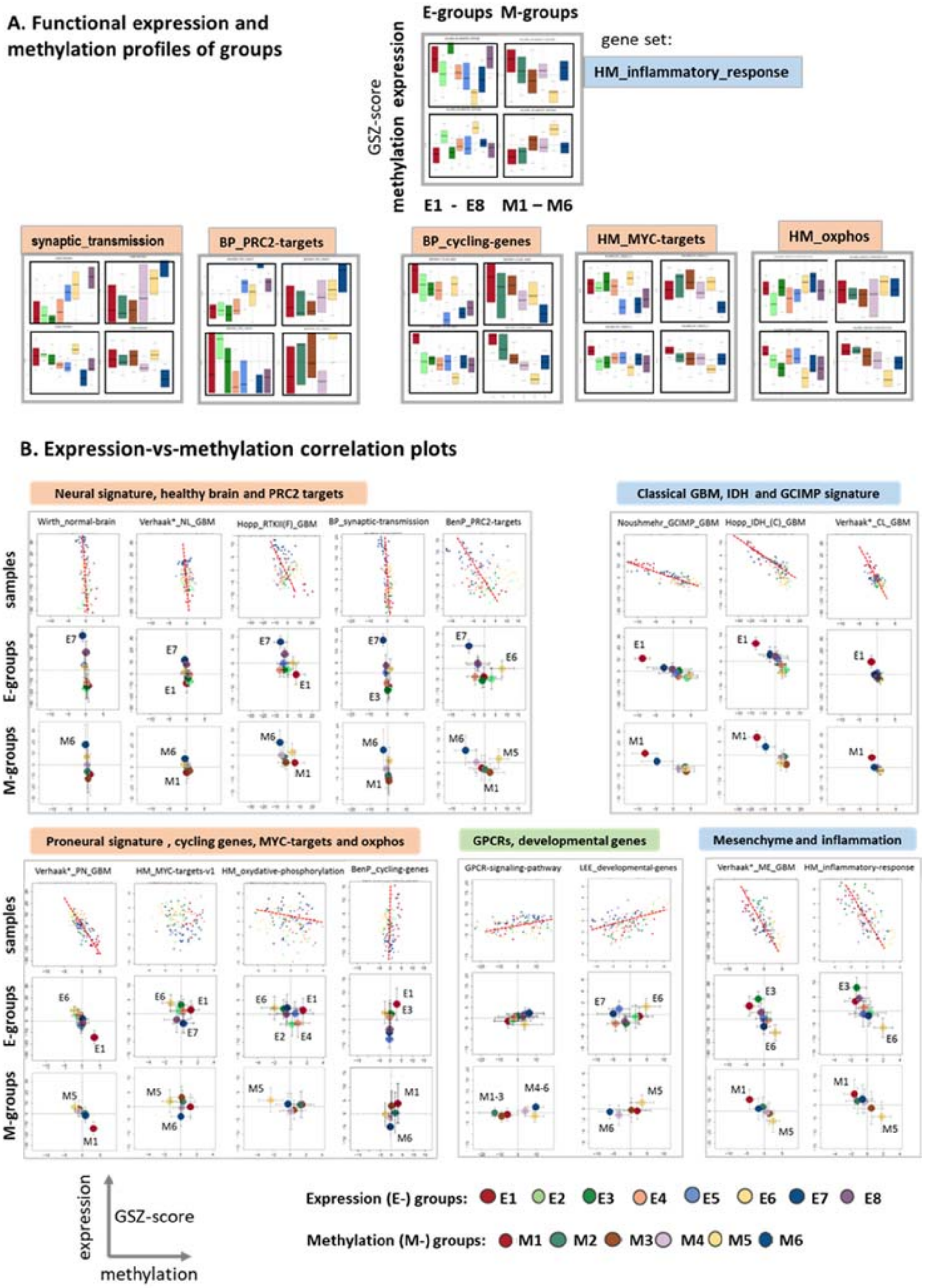

Figure S 14. Functional context of the expression and methylation GSZ-profiles of E- and M-groups: A) Each gene set signature splits into a quadruple of profiles, namely the expression and methylation signatures as revealed by E- and M-groups. Part B) shows the respective expression-versus-methylation correlation plots with sample and group resolution. Overall, the profiles of E- and M-subtypes resemble each other according to their mutual correspondence. The expression and methylation profiles are clearly anti-correlated in most cases which reflects a repressive effect of gene promoter methylation on the expression of the downstream gene on the average. The gene sets refer to different functional categories as indicated in the figure. Interestingly, the expression of G-protein coupled receptors (*GPCR*s) and of developmental genes positively correlate with methylation.

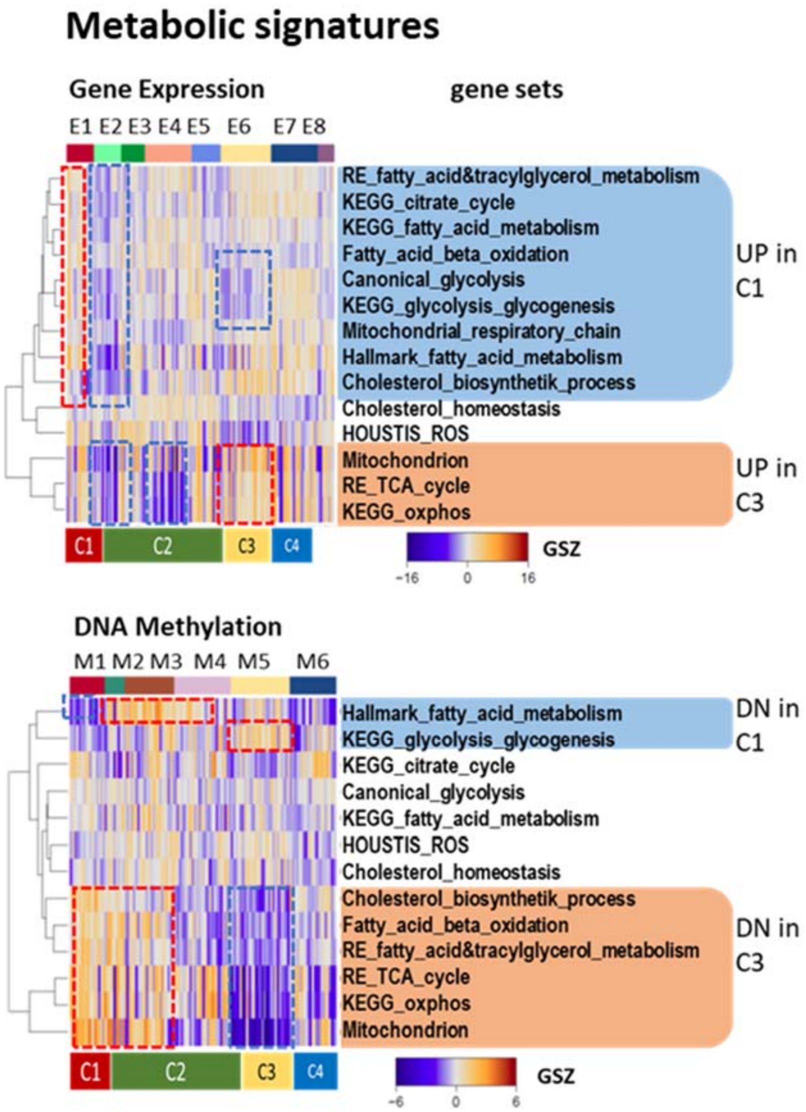

Figure S 15: Heatmaps of selected metabolic gene signatures indicate subtype-specific activation and de-activation patterns.

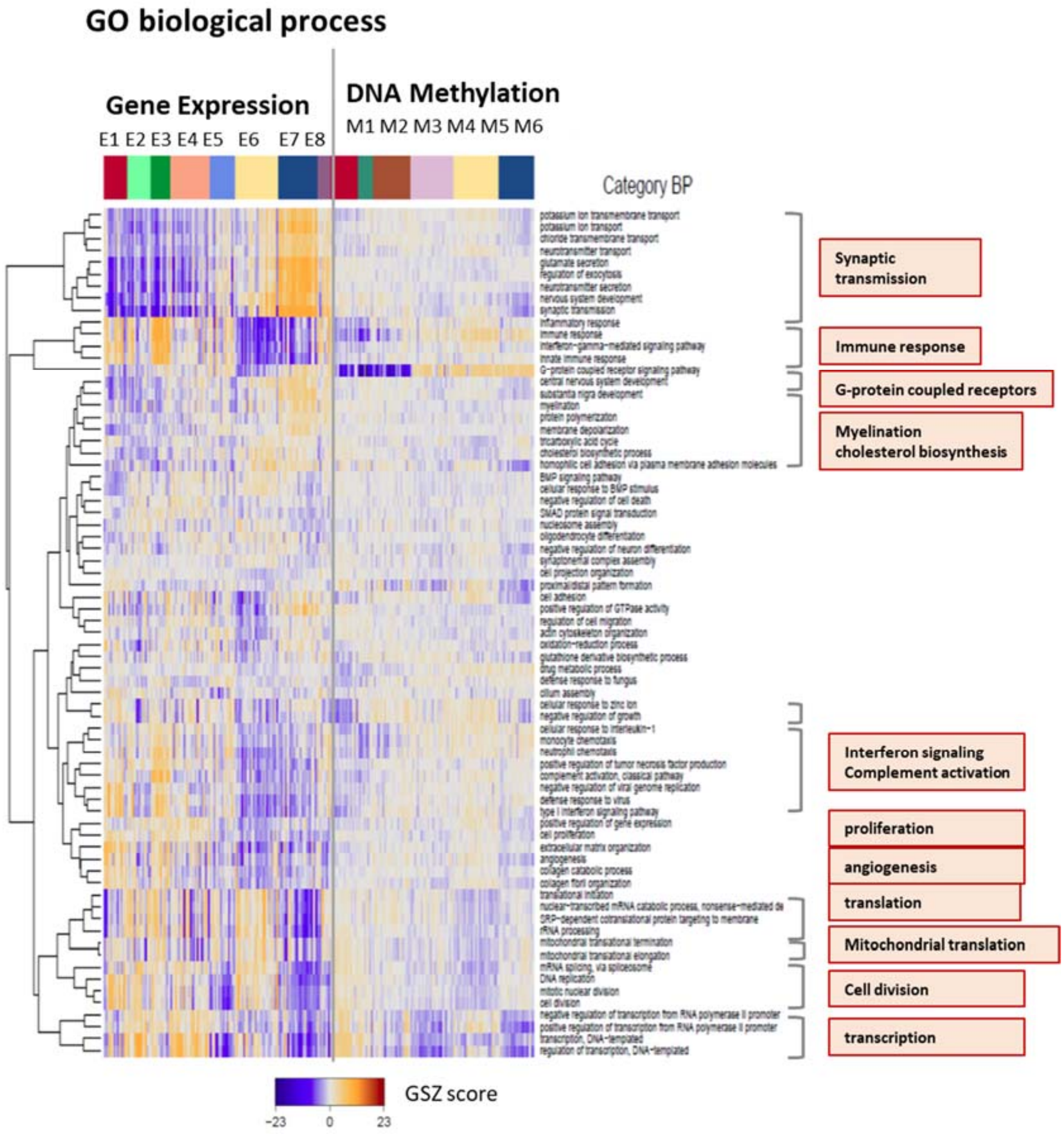
GO biological process
Gene Expression
DNA Methylation
E1 E2 E3 E4 E5 E6 E7 E8
M1 M2 M3 M4 M5 M6
Category BP
Synaptic transmission
Immune response
G-protein coupled receptors
Myelination cholesterol biosynthesis
Interferon signaling Complement activation
proliferation
angiogenesis
translation
Mitochondrial translation
Cell division
transcription
GSZ score
-23
0
23

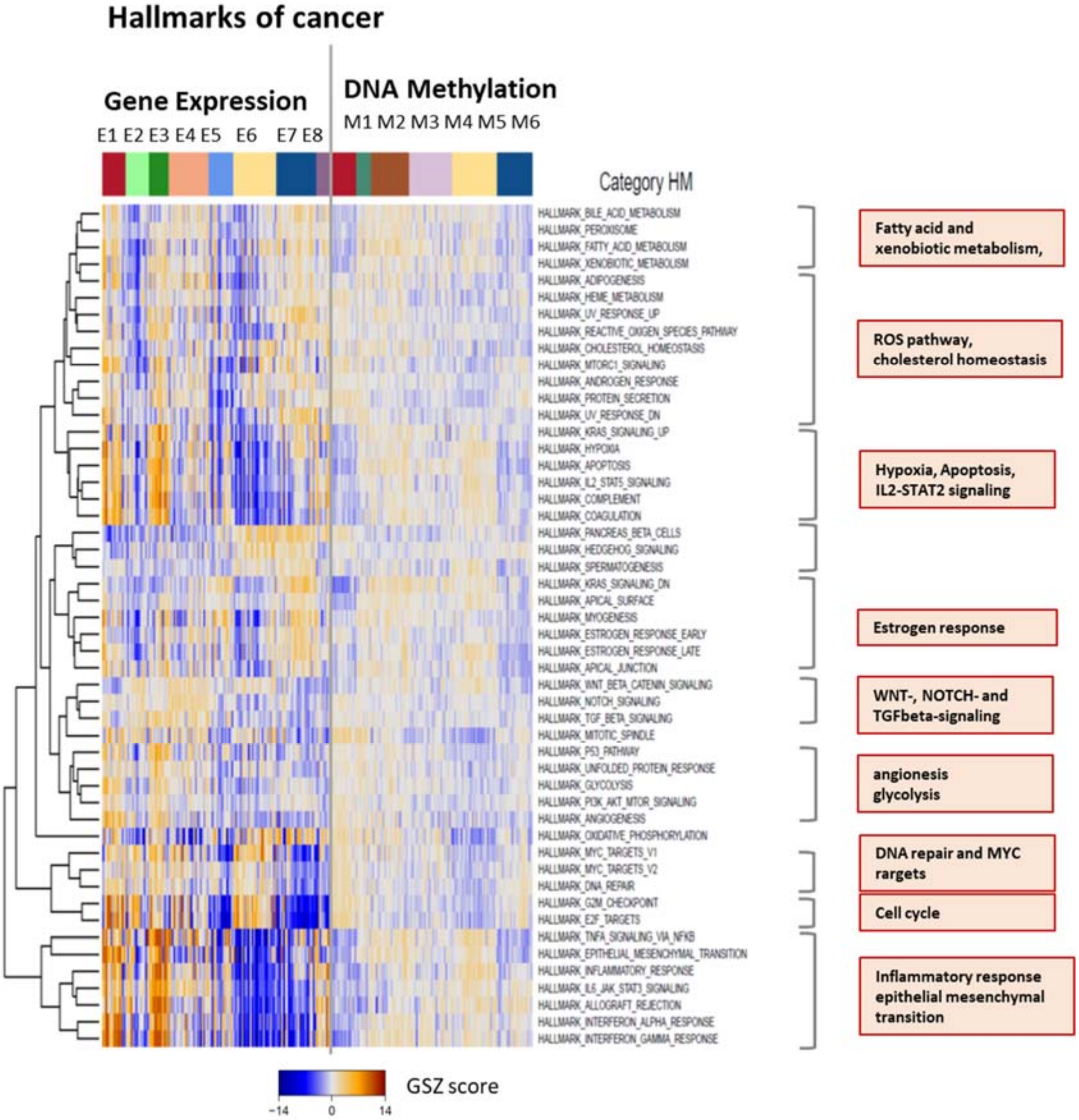

Figure S 16: Gene set analysis of functional signatures taken from the gene ontology category biological process (BP) and from the category hallmarks of cancer (HM) [10].

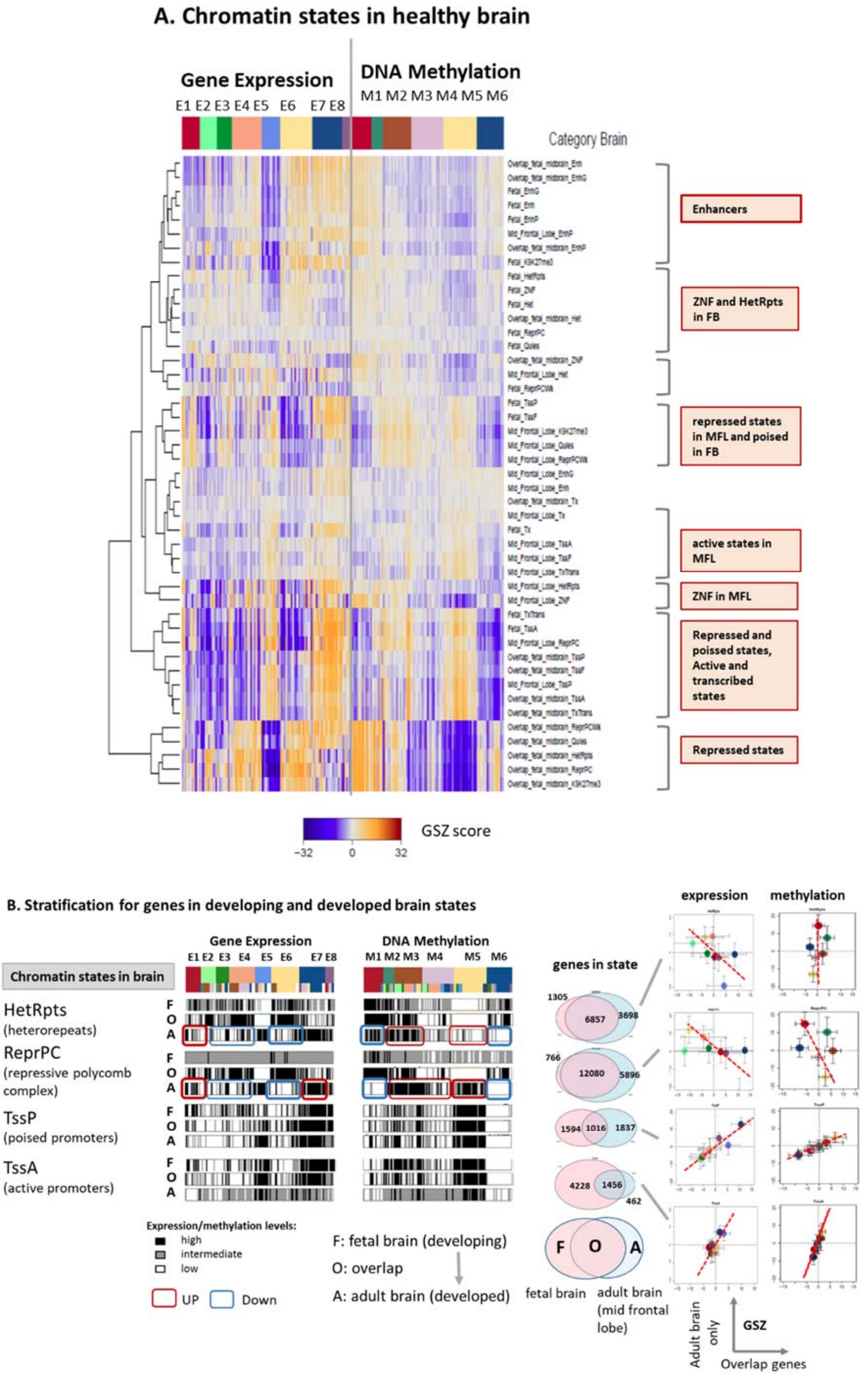
A. Chromatin states in healthy brain
Gene Expression
DNA Methylation
E1 E2 E3 E4 E5 E6 E7 E8
M1 M2 M3 M4 M5 M6
Category Brain
Overlap_fetal_midbrain_Enh
Overlap_fetal_midbrain_EnhG
Fetal_EnhG
Fetal_Enh
Fetal_EnhP
Mid_Frontal_Lobe_EnhP
Overlap_fetal_midbrain_EnhP
Fetal_K9K27me3
Fetal_HetRpts
Fetal_ZNF
Fetal_Het
Overlap_fetal_midbrain_Het
Fetal_ReprPC
Fetal_Quies
Overlap_fetal_midbrain_ZNF
Mid_Frontal_Lobe_Het
Fetal_ReprPCWk
Fetal_TssP
Fetal_TssF
Mid_Frontal_Lobe_K9K27me3
Mid_Frontal_Lobe_Quies
Mid_Frontal_Lobe_ReprPCWk
Mid_Frontal_Lobe_EnhG
Mid_Frontal_Lobe_Enh
Overlap_fetal_midbrain_Tx
Mid_Frontal_Lobe_Tx
Fetal_Tx
Mid_Frontal_Lobe_TssA
Mid_Frontal_Lobe_TssF
Mid_Frontal_Lobe_TxTrans
Mid_Frontal_Lobe_HetRpts
Mid_Frontal_Lobe_ZNF
Fetal_TxTrans
Fetal_TssA
Mid_Frontal_Lobe_ReprPC
Overlap_fetal_midbrain_TssP
Overlap_fetal_midbrain_TssF
Mid_Frontal_Lobe_TssP
Overlap_fetal_midbrain_TssA
Overlap_fetal_midbrain_TxTrans
Overlap_fetal_midbrain_ReprPCWk
Overlap_fetal_midbrain_Quies
Overlap_fetal_midbrain_HetRpts
Overlap_fetal_midbrain_ReprPC
Overlap_fetal_midbrain_K9K27me3
Enhancers
ZNF and HetRpts in FB
repressed states in MFL and poised in FB
active states in MFL
ZNF in MFL
Repressed and poissed states, Active and transcribed states
Repressed states
GSZ score
-32
0
32
B. Stratification for genes in developing and developed brain states
Gene Expression
DNA Methylation
E1 E2 E3 E4 E5 E6 E7 E8
M1 M2 M3 M4 M5 M6
Chromatin states in brain
HetRpts
(heterorepeats)
ReprPC
(repressive polycomb complex)
TssP
(poised promoters)
TssA
(active promoters)
F
O
A
Expression/methylation levels:
high
intermediate
low
UP
Down
F: fetal brain (developing)
O: overlap
A: adult brain (developed)
genes in state
1305
6857
3698
766
12080
5896
1594
1016
1837
4228
1456
462
F
O
A
fetal brain
adult brain (mid frontal lobe)
expression
methylation
Adult brain only
GSZ
Overlap genes

Figure S 17: Gene set analysis of epigenetic promoter states derived from fetal and adult healthy brain [11]. We assigned genes to promoter states as determined in [12] for developed adult brain (midfrontal lobe) and developing fetal brain tissue. Genes were divided into three categories, namely if they were found uniquely either in developing or developed brain (F- and A-genes, respectively); or together in both (overlap O-genes). The latter O-genes seem to have less impact for brain development while the F- and A-genes are assumed to be potentially related to brain development because they change state between developing and developed brain. A.) Overview heatmap of expression and methylation levels of gene promoters in the chromatin states considered (see also the glossary-list given below for assignments). Essentially one finds two major clusters referring predominantly to O-genes either in repressed or in active and poised states (two marked clusters from below). B.) For a more detailed evaluation mean expression and methylation levels of F-, O- and A-genes in selected states are shown as barcode plots. The Venn-diagrams provided their number distributions (only a relatively small number of genes is specifically repressed in the developing brain showing that repression promotes differentiation of brain tissue) and correlation plots between the expression and methylation of O- and A-genes. The patterns observed divide essentially into two types: (i) Genes in TssP and TssA states show similar expression (and methylation) profiles for F-, O- and A-genes giving rise to positive correlation between A- and O-genes. (ii) O- and A-genes in HetRpts and especially ReprPC states change their mean expression and methylation profiles especially in C3 giving rise to more puzzling, virtually negative mutual correlations. In other words, genes in repressed states with impact for brain differentiation become hypermethylated in G-CIMP-subtypes and especially in C3.

The states were defined as follows (http://egg2.wustl.edu/roadmap/web_portal/chr_state_learning.html):

1	TssP	transcription start site (TSS)_poised
2	TssF	TSS_flanking_more_upstream
3	TssA	TSS_active
4	Tx	Transcription
5	EnhG	Transcription Enhancer-like
6	Enh	Enhancer_active_with_weakK4me1_strong_K27ac
7	EnhP	Enhancer_poised
8	ReprPCWk	Repressed_polycomb_weak
9	ReprPC	Repressed_polycomb
10	K9K27me3	H3K9me3_K27me3
11	ZNF	Zinc_finger_genes_H3K36me3_K9me3
12	HetRpts	Heterochromatin_at_repeats
13	Het	Heterochromatin
14	Quies	Quiescent
15	K9acLow	low H3K9ac

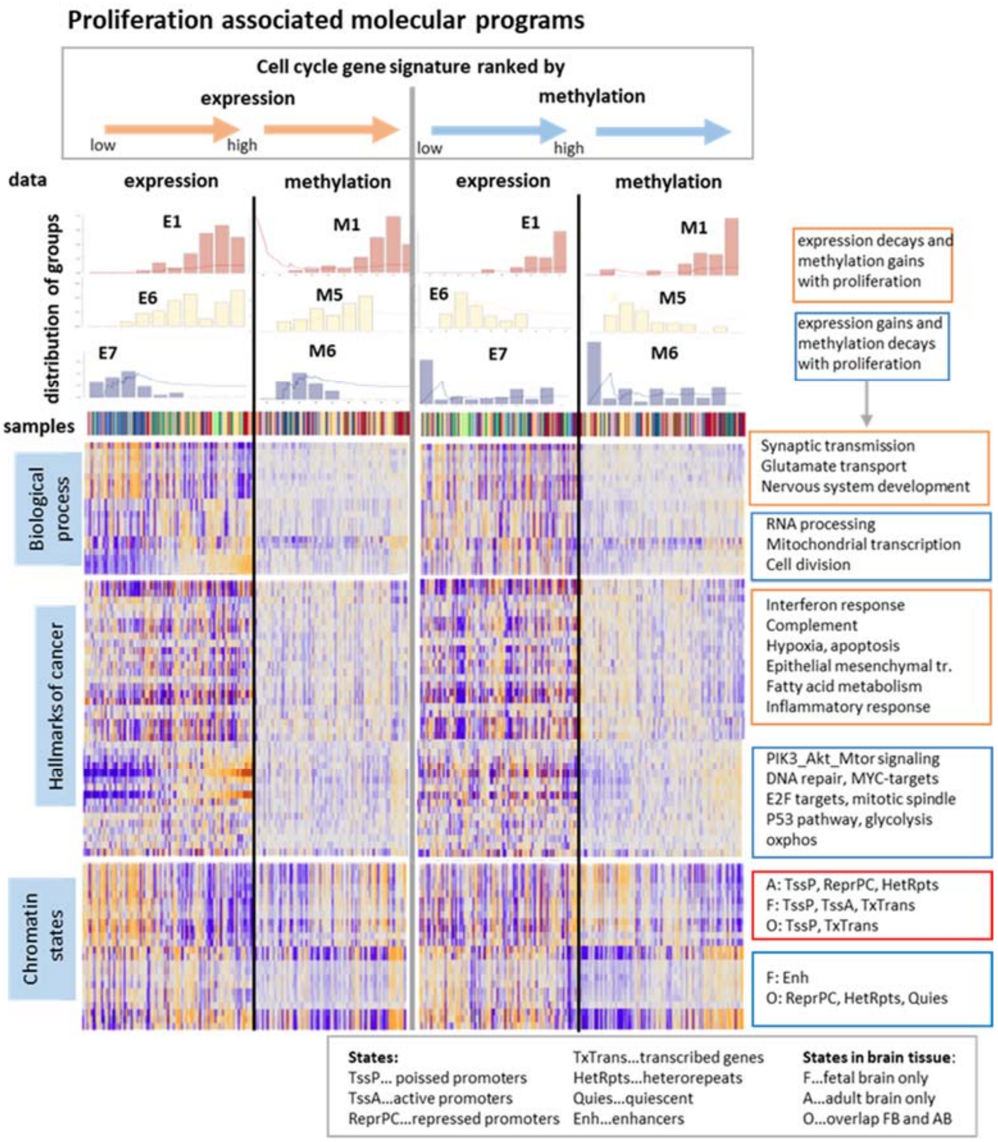

Figure S 18: Proliferation-ranked analysis of gene expression and methylation for selected functional categories and chromatin states: Tumor samples were ranked with increased expression of the gene set 'cell-cycle_literature' using the GSZ-metrics [13] (left part, see red arrows above) and with increased methylation of this set (right part, see blue arrows). The frequency distribution of selected glioma subtypes (part above) and of expression and methylation levels are shown for both rankings. One sees that tumors of the consensus class C1 (E1 and M1) associate with high expression and methylation levels of the cell-cycle related cellular program while C4 (E7 and M6) show low expression and widely distributed methylation levels. Subtype C3-tumors (E6 and M5) associate with low methylation and intermediate to high expression levels for these functions. Hence, methylation shows the opposite trend in C1 and C3 with increasing proliferative activity, namely low methylation of the cell-cycle related genes (cell division, RNA processing and mitochondrial transcription) in C3 and higher levels in C1. Transcriptional activation of cell cycle related biological processes were opposed by the decay of neuronal processes such as synaptic transmission. For methylation one finds similar parallels for processes related to inflammation which associates with the decreased immune response in C3. Interestingly, the two different patterns related to neuronal activity and inflammation on one hand (red frames) and to cell cycle activity (blue frames) associate with different chromatin states in healthy brain, namely first of all poised promoters in fetal and adult brain in the first case, which suggests that these states were affected independently of their impact for brain development. Contrarily, repressed genes in adult, developed brain show antagonistic expression and methylation compared with repressed states observed also in fetal brain. The latter ones show parallels in their methylation and, to a less degree, expression patterns with cell-cycle related processes while the former states associate with neuronal processes.

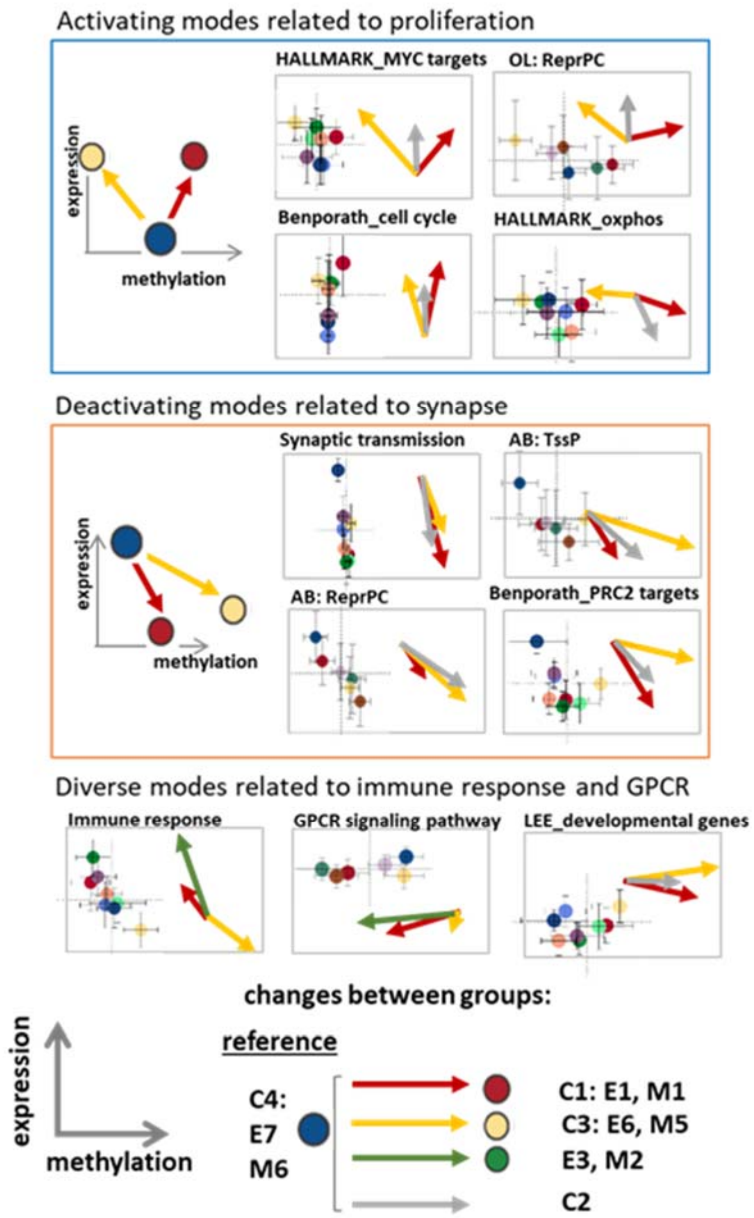

Figure S 19: Combined expression-methylation patterns: For a combined view on expression and methylation changes of selected gene sets we visualize them as arrows pointing from C4 that was chosen as ‘brain-like’ reference state towards the glioma subtypes C1, C2 and C3 in the expression-versus-methylation biplots. Overall three different combinations were identified: (i) The activating modes were related to proliferation and show increased expression which however associates either with increased (C1) or decreased (C3) methylation reflecting different driving mechanisms. (ii) Deactivating modes combine decreased expression and increased methylation in all subtypes and include functions such as synaptic transmission and a series of epigenetic signatures related to poised and repressed promoters. (iii) Functions related to immune response also show anti-correlated changes between expression and methylation but an activating effect in C1 and especially E3 and deactivating effect in C3. Hence, degeneration of healthy brain functions in all subtypes, activated proliferation in C3 and partly inflammation in E3 seem to be affected by anti-correlated DNA-promoter-methylation changes. Vector-type plots show arrows that point from C4 to C1 (red), C3 (yellow) or M2/E3 (grey) in the expression-vs-methylation plots.

A. Dimensions of glioma heterogeneity: Stratification into subtypes

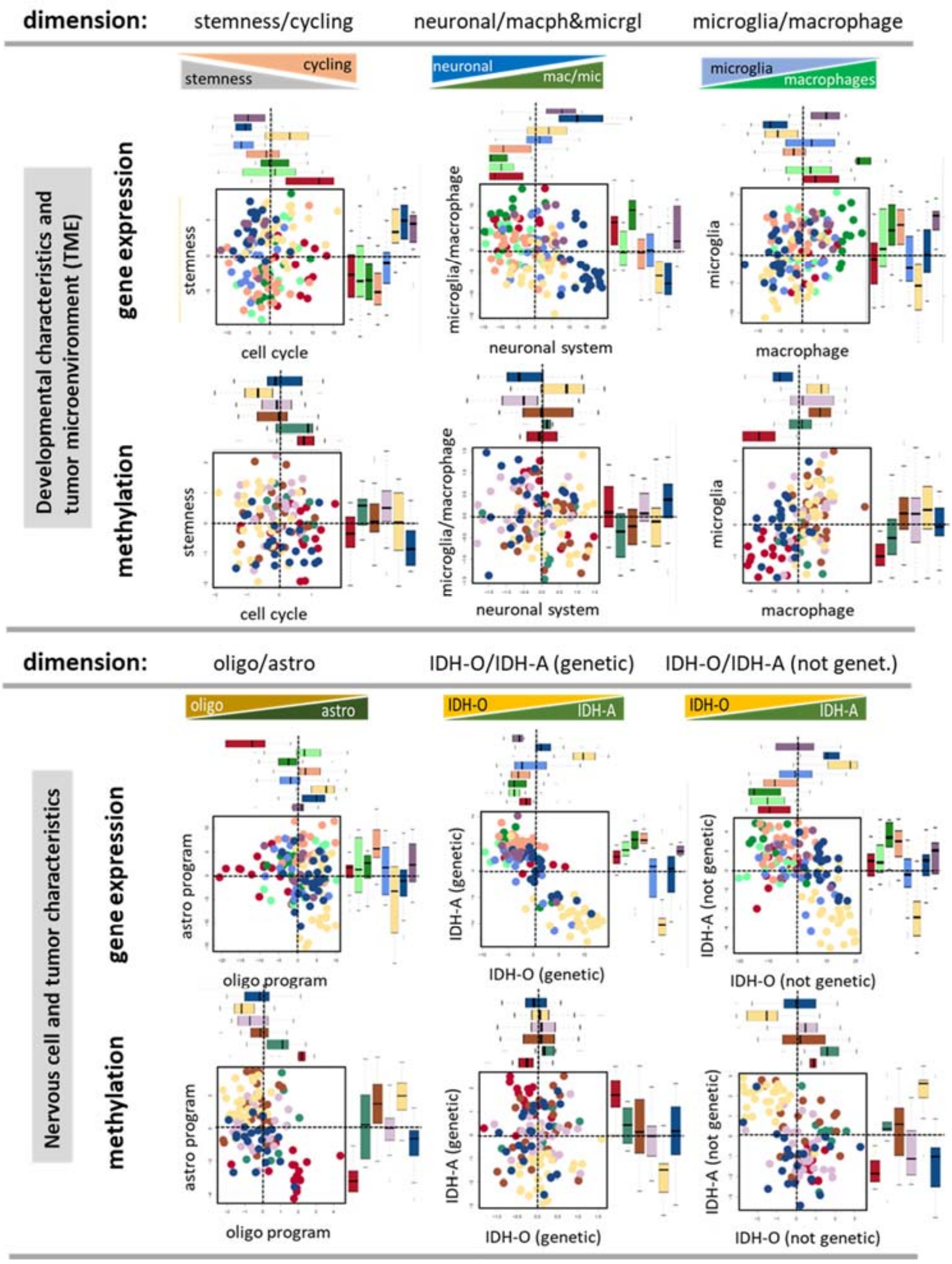

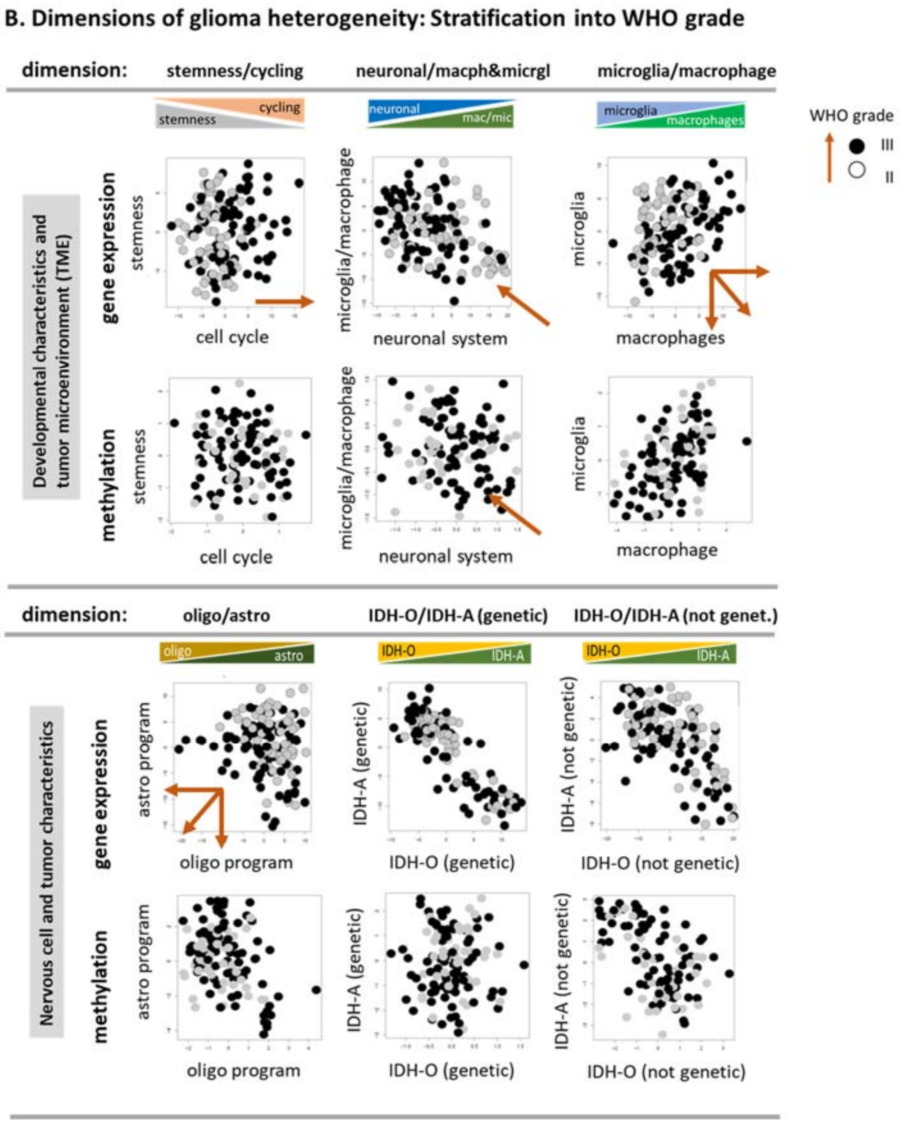

Figure S 20: A) Plots of pairwise combinations of single-cell signatures taken from [14] characterize different dimensions of glioma heterogeneity. The dots are the samples color-coded according to E1 – E8 (expression data) and M1 – M6 (methylation data) that were further summarized into barplots shown at the respective axes of the plots. Firstly, one sees that expression signatures of malignant astrocyte-like (*IDH*-A) and oligodendrocyte-like (*IDH*-O) cells are on highest levels in the C2 and C3 subtypes, respectively, as expected. E3 and E4 show the strongest activity of the *IDH*-A and astro- signatures which suggests a high content of astrocyte-like cells. Secondly, C3 and C4 (neuronal) are almost similar in their expression levels with respect to neuronal, stemness, macrophage signatures while C2 are either reduced or enhanced, respectively. Thirdly, C3 and C4 considerably differ with respect to cell cycle activity where that of C3 is close to that of C2 and C1. Fourth, anti-correlated expression and methylation patterns are found especially for the malignant *IDH*-A and *IDH*-O dimensions suggesting that neoplastic transformations in *IDH*-O and *IDH*-A cells are driven by de-methylation of the respective signature genes. Moreover, *IDH*-O and *IDH*-A expression and methylation levels change antagonistically between C1/C2 and C3 presumably due to anticorrelated amounts of *IDH*-A and *IDH*-O cell. Part B) shows the same plots as in part A however colored according to the WHO-grade (II…grey; III…black). The red arrows visualize trends of increasing WHO grade in the data. Accordingly, cell cycle and microglia/macrophage signatures gain while neuronal, astro- and oligo-signatures decline with increasing grade, on the average.

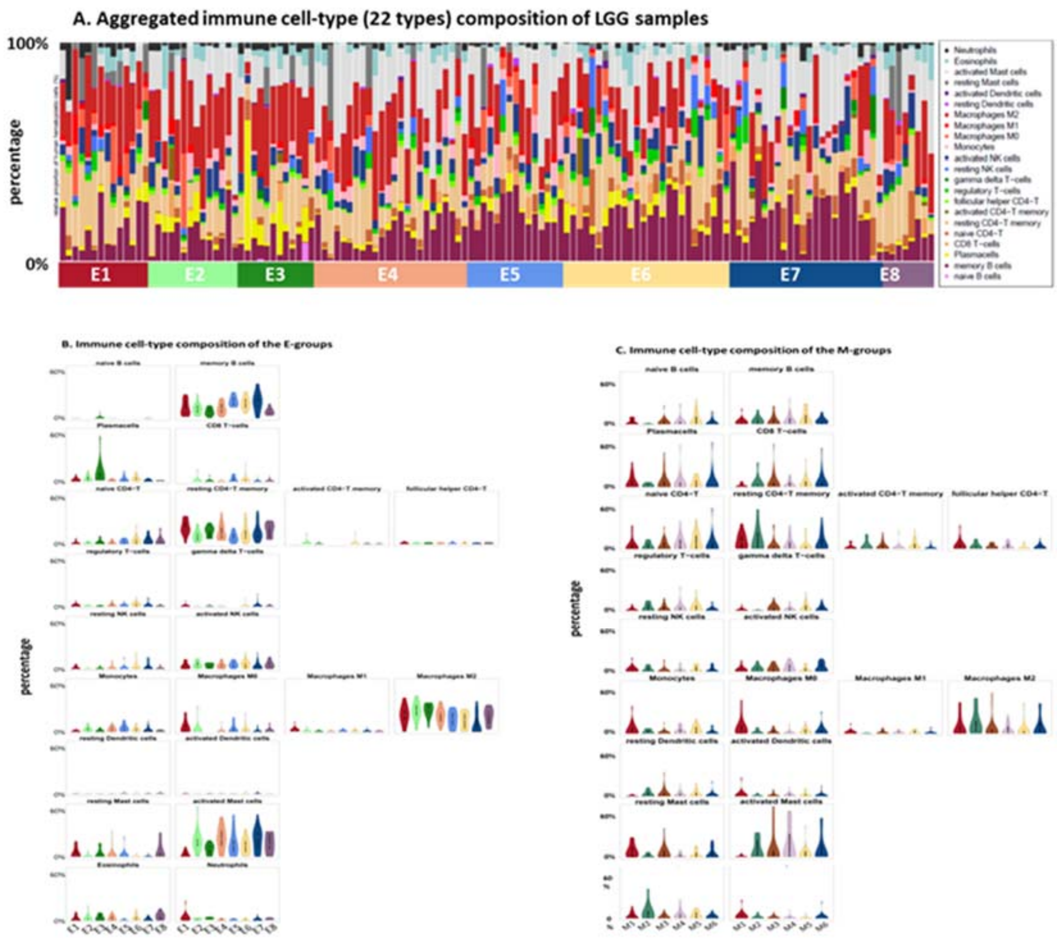

Figure S 21: Digital immune cell-type deconvolution of glioma transcriptomes using CIBERSORT [15]. The program applies support vector regression based on previous knowledge about purified leukocyte expression profiles to estimate the percentage of 22 immune cells (part A) which were further stratified into 11 cell-types (Figure 5B). Part B and C present violin plots of the compositions of the expression and methylation subgroups, respectively. Relative large amounts of macrophages are found in E1 – E3 while B-cells accumulate in E5 – E7 (part A). Interestingly, especially M2-macrophages are enriched in the astrocytic groups E1 – E4 with highest levels in E3 and E2 accumulating higher grade III tumors. M2-macrophages play a pro-tumoral role in brain cancer; they pursue an anti-inflammatory function, promote tissue remodeling and tumor growth [16]. Moreover, high levels of M2-macrophages associate with resistance to radiotherapy in grade IV GBM of the mesenchymal subtype [17]. In contrast, anti-tumoral and pro-inflammatory M1-macrophages are almost absent in all LGG subtypes studied. Large M2-macrophage abundance in astrocytic gliomas is paralleled by relatively large amounts of resting CD4-memory cells, especially in E3 and E1, and by reduced amounts of resting CD8 T-cells. The abundance of tumor infiltrating CD4+ leukocytes in GBM correlates with tumor progression and relates to tumor angiogenesis while it anti-correlates with infiltrating CD8+ leucocytes [18, 19]. We also found that activated mast cells are relatively abundant in virtually all groups of predominantly *IDH*-mutated tumors (E2 – E8), especially in E4, E5 and E7; and on relatively low level in the *IDH*-wt group E1. Mast cells were shown to become recruited and ‘educated’ by glioma cells in a glioma grade-dependent manner and reduce stemness, decrease proliferation and migration but in turn induce differentiation of glioma cells [20].

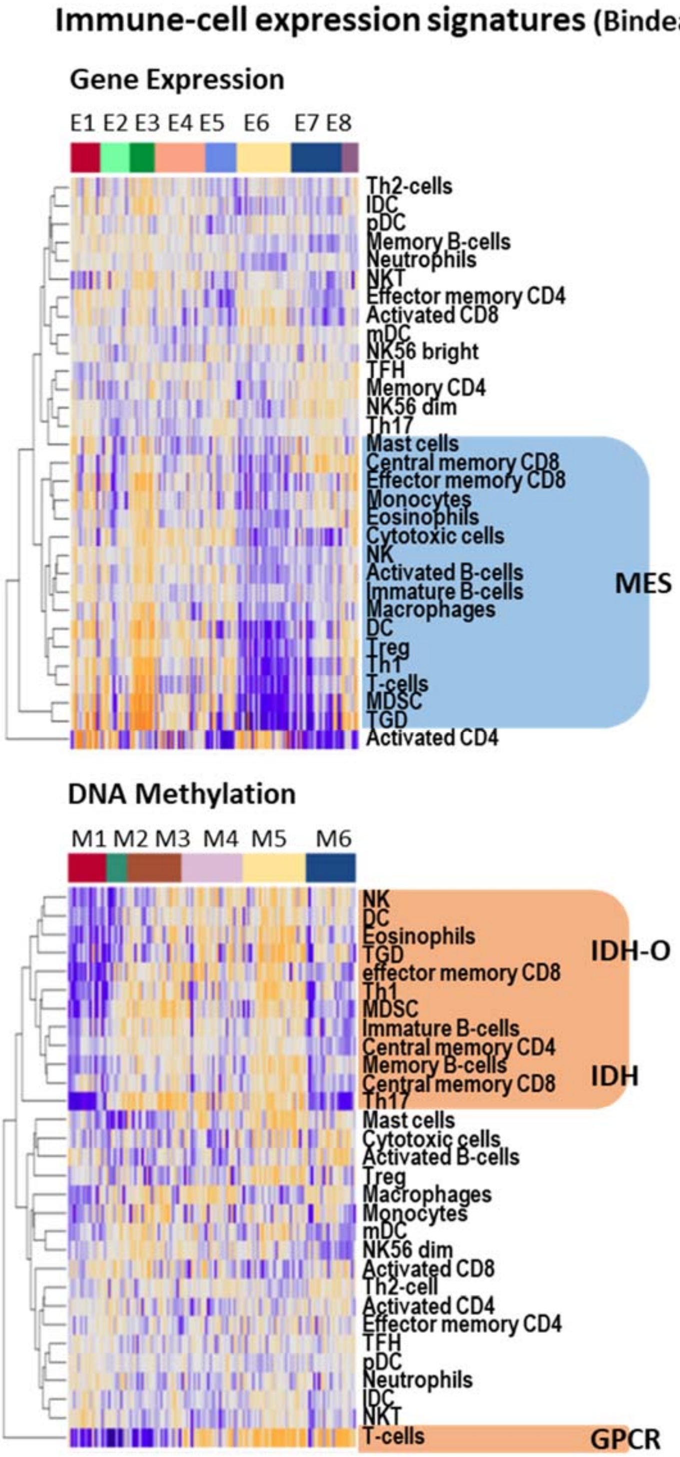

Figure S 22: Immune cell expression signatures taken from [21] confirm the signatures provided by immune cell deconvolution using CIBERSORT (Figure S 20). In contrast they estimate absolute levels of the expression signatures (versus the percentages provided by Cibersort) and they deliver also methylation profiles (part below). A series of immune cell signatures (e.g. natural killer cells (NK), effector and central memory and immature and memory B cells) reveal a G-CIMP/*IDH* and/or *IDH*-O methylation patterns while in gene expression most immune cells show a mesenchymal signature upregulated in higher grade astrocytoma (especially in E3 and E1) that supports the view that mesenchymal gliomas accumulate a series of immune cells. Interestingly, T-cells reveal a *GPCR* methylation signature and a MES expression profile. Cytotoxic cells appear activated in astrocytoma-like gliomas due to demethylation.

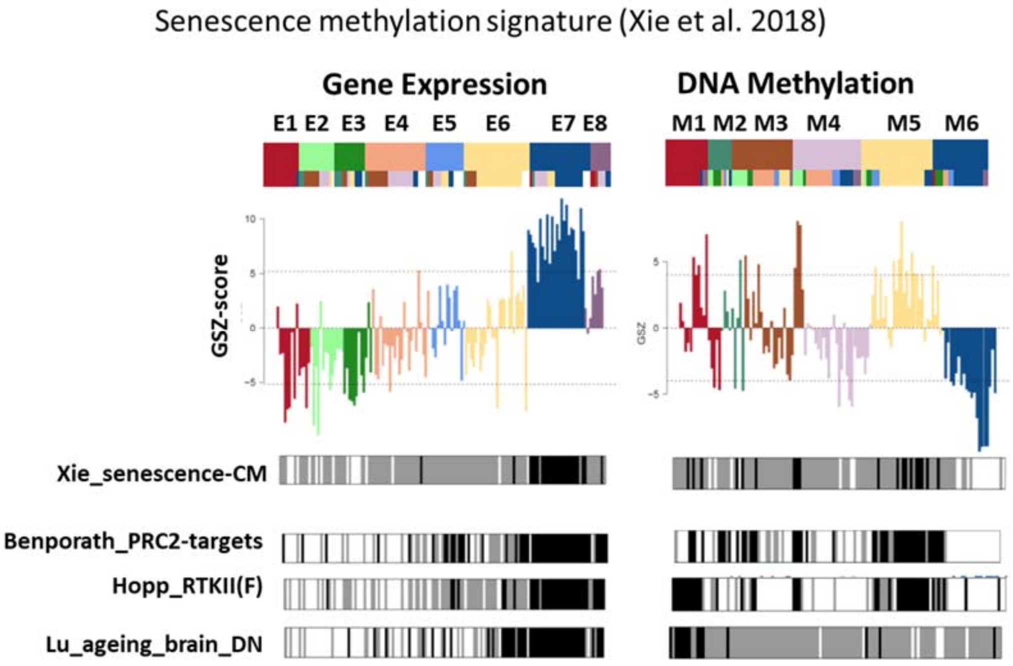

Figure S 23. The senescence methylation signature taken from [22] associates with continuously decaying expression levels of the samples in the E-groups from E7 to E1, i.e. from neuronal via pro-neural towards mesenchymal-like characteristics. This trend suggests increased senescence in parallel with tumor development and formation of a pro-inflammatory microenvironment at later stages in agreement with a recent model of glioma progression [23]. Notably, the expression and methylation profiles of the senescence signature closely resembles the profiles of the PRC2-targets and of the RTKII-methylation (GCIMP-O) signatures (see Figure 2 and Figure 3). Interestingly, the profiles of the expression signatures of the 'ageing brain and of healthy brain functions such as synaptic transmission also resemble that of the senescence signature (Figure 2 and Figure 3) while their methylation profiles differ regarding methylation of C3. It is assumed that Chr. 1p/19q-codeleted gliomas bypass senescence by other mechanisms than Chr. 1p/19q-non-codeleted tumors [23]. Our results indeed indicate that C3-tumors show increased methylation of senescence genes which however result in only moderate transcriptional deactivation compared with C1 and C2 tumors possibly because of activated oxphos-metabolism (Figure S 14) and/or deactivated inflammatory response. The 'commonly methylated' (CM) senescence signature genes become promoter-hypermethylated during aging and tumorigenesis and are thought to mediate retention of proliferating, aging cells and emerge as potential biomarkers of cancer risk [22]. They are enriched for developmental regulators which become suppressed to favor anti-differentiation and self-renewal mechanisms and eventually also for genes regulating biosynthetic and metabolic processes.

Supplementary Tables

Table S 1: List of GGN-patients whose DNA methylation data were included into the study (separate Excel-file).

Table S 2: List of TCGA-samples used for verification analyses (separate Excel-file).

Table S 3: Mutual sample distribution between expression groups defined in this publication and; A) the expression groups presented previously [24] using semi-supervised classification; B) the methylation groups of this work; C) the genomic groups presented in [24] and, D) between the methylation groups of this work and the genomic groups presented in [24]. Increased mutual overlaps are indicated by grey color.

A) Expression groups in this work versus expression groups of Weller et al. (2015) [a]										
		Expression groups (this work)								
		**E1**	**E2**	**E3**	**E4**	**E5**	**E6**	**E7**	**E8**	**Total**
Expression groups (Weller et al., 2015) [b]	**gr 1**	**11**								**11**
	**gr 2**	**3**				1	1		1	**6**
	**gr 3**							**5**		**5**
	**gr 4**		**8**	**11**		1	1		1	**22**
	**gr 5**		3		**22**					**25**
	**gr 6**		3			**12**				**15**
	**gr 7**					1	**24**	1		**26**
	**gr 8**			1	2			**18**	**6**	**27**
	**Total**	**14**	**14**	**12**	**24**	**15**	**26**	**24**	**8**	**137**

[a] Overall one finds almost a one-to-one relation between both classifications for E4 (gr. 5), E5 (gr. 6), E6 (gr. 7). Gr. 4 distributes over E2 and E3 and gr. 8 over E7 and E8 (grey fields). In turn, E1 distribute over gr.1 and 2 while E7 over gr. 3 and gr. 8. Hence, here we present a more granular classification mainly for gr.4 and gr. 8 that were split into two new strata each. On the other hand, *IDH*-wt tumors, especially of gr. 2 were assigned to strata that otherwise contain *IDH*-mut tumors.

[b] g.1- gr.3 contain exclusively *IDH*-wt tumors while gr.4- gr.8 were assigned to *IDH*-mut tumors.

B) Expression groups versus methylation groups										
		Expression groups (this work)								
		**E1**	**E2**	**E3**	**E4**	**E5**	**E6**	**E7**	**E8**	**Total**
Methylation groups (this work)	**M 1**	13						1	2	**16**
	**M 2**		2	3	1	1	1	1		**9**
	**M 3**		**6**	2	8	2	4	5	1	**28**
	**M 4**		4	1	**10**	3			3	**21**
	**M 5**		1	2		3	**18**	3		**27**
	**M 6**	1		3	2	2		**11**	2	**21**
	**Total**	**14**	**13**	**11**	**21**	**11**	**23**	**21**	**8**	**122**

C) Expression groups versus genomic groups of Weller et al. (2015) [a]										
		Expression groups (this work)								
		E1	E2	E3	E4	E5	E6	E7	E8	Total
Genomic gr.(Weller et al., 2015) [b]	gr I			2		4	24	6	1	**37**
	gr II		6	2	13	4		6		**31**
	gr III		8	8	11	6	1	7	6	**47**
	gr IV	4				1		4		**9**
	gr V	10					1	1	1	**13**
	**Total**	**14**	**13**	**11**	**21**	**11**	**23**	**21**	**8**	**122**

[a] gr. IV (*IDH*-wt and Chr7 gains) accumulate in neural E7 while gr. II (*IDH*-mut and Chr7 gains) widely distribute into E2, E4 and E7.

[b] gr.I: *IDH*-mut and Chr.1p/19q codel; gr. II: *IDH*-mut and Chr7gains; gr. III: *IDH*-mut; gr. IV: *IDH*-wt; gr. V: *IDH*-wt and Chr7 gains and Chr10 losses.

D) Methylation groups versus genomic groups of Weller et al. (2015)										
		Expression groups (this work) [a]								
		M 1	M 2	M 3	M 4	M 5	M 6			Total
Genomic groups (Weller et al., 2015)	gr I		1	3		25	3			**32**
	gr II		2	10	9		4			**25**
	gr III	2	6	10	17	1	9			**45**
	gr IV	3					4			**7**
	gr V	12					1			**13**
	**Total**	**17**	**9**	**23**	**26**	**26**	**21**			**122**

[a] gr. II (*IDH*-mut and Chr7 gains) accumulate in M3

Table S 4: Distribution of cases with distinct genetic and clinical characteristics in expression groups ('*' indicates significant enrichment with p<0.05 according to Fisher's exact test)

		Ex1	***Ex2***	***Ex3***	***Ex4***	***Ex5***	***Ex6***	***Ex7***	***Ex8***
Total	number of samples	14	14	12	24	15	26	24	8
		10%	10%	9%	18%	11%	19%	18%	6%
IDH 1/2 status	wildtype	**14***				1	1	5	1
	mutated		14	12	**24***	14	**25***	19	7
Chr1p19q	intact	**14***	12	10	**23***	11	2	18	7
	codel	0	2	2	1	4	**24***	6	1
Chr7	gain	**10***	4	2	8	5	4	4	1
	normal	3	10	10	16	10	22	20	7
Chr10	loss	**10***	2	2	1	4	2	3	2
	normal	4	12	10	**23**	11	24	21	6
Chr1	loss	0	2	2	1	4	24	6	1
	normal	**14***	12	10	**23***	11	2	18	7
Chr19	loss	2	5	4	4	6	**25***	7	2
	normal	**12***	9	8	**20***	9	1	17	6
TERT status	mutated	**8***	1	0	1	4	**24***	5	2
	wildtype	4	**12***	**11***	**23***	10	2	19	6
MGMT status	methylated	6	13	11	20	13	24	18	6
	non methylated	**8***	1	1	4	1	2	6	2
Grade	III	12	10	**12***	10	4	**23***	11	7
	II	2	4	0	**14***	**11***	3	13	1
Histology	O	1	5	7	1	2	**22***	8	4
	A	**13***	9	5	**23***	**13***	4	16	4
Gender	male	5	9	7	15	9	13	18	**8***
	female	**9***	5	5	9	6	13	6	0

Table S 5: Distribution of cases with distinct genetic and clinical characteristics in methylation groups ('*' indicates significant enrichment with p<0.05 acording to Fisher's exact test)

		M1	*M2*	*M3*	*M4*	*M5*	*M6*
Total	number of samples	16	9	23	26	27	21
IDH 1/2 status	wildtype	**14***	0	0	0	0	5
	mutated	2	9	**23***	**26***	**27***	16
Chr1p19q	intact	**16***	7	**20***	**25***	0	18
	codel	0	2	3	1	**27***	3
Chr7	gain	**12***	2	10	5	1	4
	normal	4	7	13	21	**26***	17
Chr10	loss	**11***	2	4	2	0	5
	normal	5	7	19	24	**27***	16
Chr1	loss	0	2	3	1	**27***	3
	normal	**16***	7	**20***	**25***	0	18
Chr19	loss	2	3	9	4	**27***	6
	normal	**14***	6	14	**22***	0	15
TERT status	mutated	**9***	1	4	1	**23***	2
	wildtype	5	8	**19***	**24***	3	**19***
MGMT status	methylated	8	9	20	24	**26***	14
	non methylated	**8***	0	3	2	1	6
Grade	III	13	7	16	14	22	10
	II	3	2	7	12	5	**11***
Histology	O	1	5	7	6	**22***	4
	A	**15***	4	16	20	5	**17***
Gender	male	7	6	11	**22***	16	12
	female	9	3	12	4	11	9

Table S 6: Glioma gene sets

**Short name**	**Comment**	**Data set**	**Reference**
**Expression signatures**			
Verhaak* (reanalyzed by Hopp et al.)	Four GBM-subtypes: CL, MES, PN, NL and healthy brain	TCGA, 153 GBM and 10 normal	[25]; [26]
Reifenberger	For GBM-subtypes: CL, MES, PN-wt (*IDH*1/2) and PN-mut	GGN, 94 GBM	[9]
Donson	STS and LTS with inflammatory signature of LTS	26 GBM (mostly pediatric) and AA	[27]
Dong	GBM versus healthy brain	TCGA, 240 GBM and 10 normal	[28]
Weller	Eight expression subtypes E1 – E8	GGN, 137 LGG	[24]
Mukasa	Histological classes and 1p/19q	Grade II-V, 21 gliomas	[29]
Gorovets	Three LGG subtypes: PG (pre-GBM), EPL and NP	101 grade II and III gliomas	[30]
**Combined signature**			
Noushmehr	G-CIMP signature of genes hypermethylated AND underexpressed in *IDH* mutated GBM	TCGA, 272 GBM	[31]
**Methylation signatures**			
Laffaire	Diverse methylation signatures	33 LGG and 36 GBM	[32]
Christensen	Gliomas of grade II and III	131 grade II-IV gliomas	[33]
Sturm/Hopp (reanalyzed by Hopp et al.)	Six GBM-subtypes: RTKI, RTKII, MS, *IDH*, G34 and K27	TCGA-GBM including pediatric GBM	[4]; [34]
Shinawi			[35]
Martinez	Methylation signatures	87 GBM	[36]
Wirth	Healthy brain tissue		[37]
**Glioma single cell signatures**			
Venteicher	Single cell transcriptomics, cell-type signatures of astrocytes, oligodendrocytes, microglia/macrophages and of derived malignant programs		[14]
**Glioma single treatment resistance signature**			

Segerman	The signature was derived from libraries of glioma-initiating cell clones from glioblastoma (*IDH*-wt) patient samples considering a range of responses to radiation and drugs		[38]

References

1. Gerber T, Willscher E, Loeffler-Wirth H, Hopp L, Schadendorf D, Schartl M, Anderegg U, Camp G, Treutlein B, Binder H, Kunz M: **Mapping heterogeneity in patient-derived melanoma cultures by single-cell RNA-seq.** *Oncotarget* 2016, **8:**846-862.
2. Glusman G, Yanai I, Rubin I, Lancet D: **The Complete Human Olfactory Subgenome.** *Genome Research* 2001, **11:**685-702.
3. Lessin SR, Huebner K, Isobe M, Croce CM, Steinert PM: **Chromosomal Mapping of Human Keratin Genes: Evidence of Non-linkage.** *Journal of Investigative Dermatology* 1988, **91:**572-578.
4. Hopp L, Willscher E, Wirth-Loeffler H, Binder H: **Function Shapes Content: DNA-Methylation Marker Genes and their Impact for Molecular Mechanisms of Glioma.** *Journal of Cancer Research Updates* 2015, **4:**127-148.
5. Kunz M, Löffler-Wirth H, Dannemann M, Willscher E, Doose G, Kelso J, Kottek T, Nickel B, Hopp L, Landsberg J, et al: **RNA-seq analysis identifies different transcriptomic types and developmental trajectories of primary melanomas.** *Oncogene* 2018.
6. Ceccarelli M, Barthel Floris P, Malta Tathiane M, Sabedot Thais S, Salama Sofie R, Murray Bradley A, Morozova O, Newton Y, Radenbaugh A, Pagnotta Stefano M, et al: **Molecular Profiling Reveals Biologically Discrete Subsets and Pathways of Progression in Diffuse Glioma.** *Cell* 2016, **164:**550-563.
7. Paul Y, Mondal B, Patil V, Somasundaram K: **DNA methylation signatures for 2016 WHO classification subtypes of diffuse gliomas.** *Clinical epigenetics* 2017, **9:**32-32.
8. de Souza CF, Sabedot TS, Malta TM, Stetson L, Morozova O, Sokolov A, Laird PW, Wiznerowicz M, Iavarone A, Snyder J, et al: **A Distinct DNA Methylation Shift in a Subset of Glioma CpG Island Methylator Phenotypes during Tumor Recurrence.** *Cell Reports* 2018, **23:**637-651.
9. Reifenberger G, Weber RG, Riehmer V, Kaulich K, Willscher E, Wirth H, Gietzelt J, Hentschel B, Westphal M, Simon M, et al: **Molecular characterization of long-term survivors of glioblastoma using genome- and transcriptome-wide profiling.** *International Journal of Cancer* 2014, **135:**1822-1831.
10. Liberzon A, Birger C, Thorvaldsdóttir H, Ghandi M, Mesirov Jill P, Tamayo P: **The Molecular Signatures Database Hallmark Gene Set Collection.** *Cell Systems* 2015, **1:**417-425.
11. Roadmap Epigenomics Consortium, Kundaje A, Meuleman W, Ernst J, Bilenky M, Yen A, Heravi-Moussavi A, Kheradpour P, Zhang Z, Wang J, et al: **Integrative analysis of 111 reference human epigenomes.** *Nature* 2015, **518:**317-330.
12. Ernst J, Kellis M: **Discovery and characterization of chromatin states for systematic annotation of the human genome.** *Nat Biotech* 2010, **28:**817-825.
13. Whitfield ML, Sherlock G, Saldanha AJ, Murray JI, Ball CA, Alexander KE, Matese JC, Perou CM, Hurt MM, Brown PO, Botstein D: **Identification of Genes Periodically Expressed in the Human Cell Cycle and Their Expression in Tumors.** *Molecular Biology of the Cell* 2002, **13:**1977-2000.
14. Venteicher AS, Tirosh I, Hebert C, Yizhak K, Neftel C, Filbin MG, Hovestadt V, Escalante LE, Shaw ML, Rodman C, et al: **Decoupling genetics, lineages, and microenvironment in IDH-mutant gliomas by single-cell RNA-seq.** *Science* 2017, **355**.
15. Newman AM, Liu CL, Green MR, Gentles AJ, Feng W, Xu Y, Hoang CD, Diehn M, Alizadeh AA: **Robust enumeration of cell subsets from tissue expression profiles.** *Nature Methods* 2015, **12:**453.
16. Roesch S, Rapp C, Dettling S, Herold-Mende C: **When Immune Cells Turn Bad—Tumor-Associated Microglia/Macrophages in Glioma.** *International Journal of Molecular Sciences* 2018, **19:**436.
17. Wang Q, Hu B, Hu X, Kim H, Squatrito M, Scarpace L, deCarvalho AC, Lyu S, Li P, Li Y, et al: **Tumor Evolution of Glioma-Intrinsic Gene Expression Subtypes Associates with Immunological Changes in the Microenvironment.** *Cancer Cell* 2017, **32:**42-56.e46.

18. Han S, Zhang C, Li Q, Dong J, Liu Y, Huang Y, Jiang T, Wu A: **Tumour-infiltrating CD4+ and CD8+ lymphocytes as predictors of clinical outcome in glioma.** *British Journal Of Cancer* 2014, **110:**2560.
19. Mu L, Yang C, Gao Q, Long Y, Ge H, DeLeon G, Jin L, Chang Y, Sayour EJ, Ji J, et al: **CD4+ and Perivascular Foxp3+ T Cells in Glioma Correlate with Angiogenesis and Tumor Progression.** *Frontiers in Immunology* 2017, **8:**1451.
20. Attarha S, Roy A, Westermark B, Tchougounova E: **Mast cells modulate proliferation, migration and stemness of glioma cells through downregulation of GSK3β expression and inhibition of STAT3 activation.** *Cellular Signalling* 2017, **37:**81-92.
21. Bindea G, Mlecnik B, Tosolini M, Kirilovsky A, Waldner M, Obenauf AC: **Spatiotemporal dynamics of intratumoral immune cells reveal the immune landscape in human cancer.** *Immunity* 2013, **39:**782 - 795.
22. Xie W, Kagiampakis I, Pan L, Zhang YW, Murphy L, Tao Y, Kong X, Kang B, Xia L, Carvalho FLF, et al: **DNA Methylation Patterns Separate Senescence from Transformation Potential and Indicate Cancer Risk.** *Cancer Cell* 2018, **33:**309-321.e305.
23. Barthel FP, Wesseling P, Verhaak RGW: **Reconstructing the molecular life history of gliomas.** *Acta Neuropathologica* 2018, **135:**649-670.
24. Weller M, Weber R, Willscher E, Riehmer V, Hentschel B, Kreuz M, Felsberg J, Beyer U, Löffler-Wirth H, Kaulich K, et al: **Molecular classification of diffuse cerebral WHO grade II/III gliomas using genome- and transcriptome-wide profiling improves stratification of prognostically distinct patient groups.** *Acta Neuropathologica* 2015:1-15.
25. Hopp L, Wirth H, Fasold M, Binder H: **Portraying the expression landscapes of cancer subtypes: A glioblastoma multiforme and prostate cancer case study.** *Systems Biomedicine* 2013, **1:**99-121.
26. Verhaak RGW, Hoadley KA, Purdom E, Wang V, Qi Y, Wilkerson MD, Miller CR, Ding L, Golub T, Mesirov JP, et al: **Integrated Genomic Analysis Identifies Clinically Relevant Subtypes of Glioblastoma Characterized by Abnormalities in PDGFRA, IDH1, EGFR, and NF1.** *Cancer Cell* 2010, **17:**98-110.
27. Donson AM, Birks DK, Schittone SA, Kleinschmidt-DeMasters BK, Sun DY, Hemenway MF, Handler MH, Waziri AE, Wang M, Foreman NK: **Increased Immune Gene Expression and Immune Cell Infiltration in High-Grade Astrocytoma Distinguish Long-Term from Short-Term Survivors.** *The Journal of Immunology* 2012, **189:**1920-1927.
28. Dong H, Siu H, Luo L, Fang X, Jin L, Xiong M: **Investigation gene and microRNA expression in glioblastoma.** *BMC Genomics* 2010, **11:**S16.
29. Mukasa A, Ueki K, Ge X, Ishikawa S, Ide T, Fujimaki T, Nishikawa R, Asai A, Kirino T, Aburatani H: **Selective Expression of a Subset of Neuronal Genes in Oligodendroglioma with Chromosome 1p Loss.** *Brain Pathology* 2004, **14:**34-42.
30. Gorovets D, Kannan K, Shen R, Kastenhuber ER, Islamdoust N, Campos C, Pentsova E, Heguy A, Jhanwar SC, Mellinghoff IK, et al: **IDH Mutation and Neuroglial Developmental Features Define Clinically Distinct Subclasses of Lower Grade Diffuse Astrocytic Glioma.** *Clinical Cancer Research* 2012, **18:**2490-2501.
31. Noushmehr H, Weisenberger DJ, Diefes K, Phillips HS, Pujara K, Berman BP, Pan F, Pelloski CE, Sulman EP, Bhat KP, et al: **Identification of a CpG Island Methylator Phenotype that Defines a Distinct Subgroup of Glioma.** *Cancer Cell* 2010, **17:**510-522.
32. Laffaire J, Everhard S, Idbaih A, Crinière E, Marie Y, de Reyniès A, Schiappa R, Mokhtari K, Hoang-Xuan K, Sanson M, et al: **Methylation profiling identifies 2 groups of gliomas according to their tumorigenesis.** *Neuro-Oncology* 2011, **13:**84-98.
33. Christensen BC, Smith AA, Zheng S, Koestler DC, Houseman EA, Marsit CJ, Wiemels JL, Nelson HH, Karagas MR, Wrensch MR, et al: **DNA Methylation, Isocitrate Dehydrogenase Mutation, and Survival in Glioma.** *Journal of the National Cancer Institute* 2011, **103:**143-153.

34. Sturm D, Witt H, Hovestadt V, Khuong-Quang D-A, Jones David TW, Konermann C, Pfaff E, Tönjes M, Sill M, Bender S, et al: **Hotspot Mutations in H3F3A and IDH1 Define Distinct Epigenetic and Biological Subgroups of Glioblastoma.** *Cancer Cell* 2012, **22:**425-437.
35. Shinawi T, Hill VK, Krex D, Schackert G, Gentle D, Morris MR, Wei W, Cruickshank G, Maher ER, Latif F: **DNA methylation profiles of long- and short-term glioblastoma survivors.** *Epigenetics* 2013, **8:**149-156.
36. Martinez R, Martin-Subero JI, Rohde V, Kirsch M, Alaminos M, Fernández AF, Ropero S, Schackert G, Esteller M: **A microarray-based DNA methylation study of glioblastoma multiforme.** *Epigenetics* 2009, **4:**255-264.
37. Wirth H, Löffler M, von Bergen M, Binder H: **Expression cartography of human tissues using self organizing maps.** *BMC Bioinformatics* 2011, **12:**306.
38. Segerman A, Niklasson M, Haglund C, Bergström T, Jarvius M, Xie Y, Westermark A, Sönmez D, Hermansson A, Kastemar M, et al: **Clonal Variation in Drug and Radiation Response among Glioma-Initiating Cells Is Linked to Proneural-Mesenchymal Transition.** *Cell Reports* 2016, **17:**2994-3009.
